# Reducing the basic reproduction number of COVID-19: a model simulation focused on QALYs, hospitalisation, productivity costs and optimal (soft) lockdown

**DOI:** 10.1007/s10198-022-01500-7

**Published:** 2022-08-02

**Authors:** Jose Robles-Zurita

**Affiliations:** 1 Health Economics and Health Technology Assessment, School of Health & Wellbeing, University of Glasgow, Glasgow, United Kingdom; 2 HCD Economics, Daresbury, United Kingdom

**Keywords:** COVID-19, Lockdown, QALYs, Hospitalisation, Productivity costs, I18

## Abstract

Even if public health interventions are successful at reducing the spread of COVID-19, there is no guarantee that they will bring net benefits to the society because of the dynamic nature of the pandemic, e.g., the risk of a second outbreak if those interventions are stopped too early, and the costs of a continued lockdown. In this analysis, a discrete-time dynamic model is used to simulate the effect of reducing the effective reproduction number, driven by lockdowns ordered in March 2020 in four European countries (UK, France, Italy and Spain), on QALYs and hospitalisation costs. These benefits are valued in monetary terms (€30,000 per QALY assumed) and compared to productivity costs due to reduced economic activity during the lockdown. An analysis of the optimal duration of lockdown is performed where a net benefit is maximised. The switch to a soft lockdown is analysed and compared to a continued lockdown or no intervention. Results vary for two assumptions about hospital capacity of the health system: (a) under unlimited capacity, average benefit ranges from 8.21 to 14.21% of annual GDP, for UK and Spain, respectively; (b) under limited capacity, average benefits are higher than 30.32% of annual GDP in all countries. The simulation results imply that the benefits of lockdown are not substantial unless continued until vaccination of high-risk groups is complete. It is illustrated that lockdown may not bring net benefits under some scenarios and a soft lockdown will be a more efficient alternative from mid-June 2020 only if the basic reproduction number is maintained low (not necessarily below 1) and productivity costs are sufficiently reduced.

**Supplementary Information:**

The online version contains supplementary material available at 10.1007/s10198-022-01500-7.

## Introduction

The COVID-19 pandemic has created a situation where decisions must be made in the absence of key information about the effectiveness of interventions. A need for models to assess the link between interventions and their consequences is apparent. While epidemiological models are often built to predict intermediate outcomes (like final size of epidemic, deaths or healthcare demand), a health economic appraisal requires the analysis of the value of policies being evaluated during the current crisis [1–3]. Decision modelling should focus on final benefits and costs of different courses of action. A discussion is in place on the need to translate intermediate outcomes to comprehensive measures of health like the quality-adjusted life year (QALY).^1^ Furthermore, the use of monetary values of QALYs is an extended practice in health economics and allows the decision maker to compare health benefits with consequences in different dimensions of life [4, 5]. This is important since interventions aimed at reducing the burden of the COVID-19 pandemic have consequences across different sectors.

In this paper, a discrete-time dynamic model is presented to simulate the effect of changes in the basic reproduction number of COVID-19 on QALYs saved and hospitalisation costs avoided. This model was used to illustrate the evaluation of lockdown interventions in four European countries (UK, France, Italy and Spain). Values of model parameters are based on available evidence and information from national statistics. In addition, assumptions regarding the impact of hospital capacity on COVID-19 prognosis, duration of lockdown and time to availability of a vaccine are modified to compare different scenarios. An analysis of optimal lockdown duration is performed by comparing benefits, i.e., monetary value of QALYs saved and hospitalisation costs avoided, to gross domestic product (GDP) losses over time in lockdown. Finally, characteristics of soft lockdown, in terms of basic reproduction number and productivity costs, are analysed to represent an improved alternative compared to continued full lockdown or no intervention.

The results indicate that considering different scenarios and incorporating the time dimension of lockdown measures in a dynamic fashion is important to evaluate public health interventions affecting epidemiological parameters like the basic reproduction number. Alternatives to hard lockdown can be more efficient only if the basic reproduction number is kept low (not necessarily below one) and productivity losses are sufficiently reduced.

## Methods

### Model

An age-stratified discrete-time dynamic model is used which follows the logic of epidemiological SIR (susceptible-infected-recovered) models, dividing the population into susceptible, infected and recovered [6, 7]. In addition, people may die due to background or COVID-19-related mortality. The pandemic starts at time $t=1$ with an initial infection $I\left(t=1\right)=1$ in a closed population of size $P$ divided into $N$ age groups with size ${P}^{G}$ for group $G$. In the model, $I(t)$ includes only new infections at period $t$, which are secondary infections produced by the new infections in the previous period $I(t-1)$. Therefore, an infected person produces all its secondary infections over the course of one time period, where a time unit represents the serial interval, i.e., the estimated average time for an infection case to produce its secondary infections [8]. Infected people can suffer either no/mild or severe symptoms; a proportion of those with severe symptoms will die due to COVID-19.

The progression of the pandemic is determined by the basic reproduction number ${R}_{0}(t)$ at each time $t$, defined as the number of secondary infections produced by an infected person assuming a fully susceptible population [9]. Only a proportion ${p}^{G}$ of the basic reproduction number ${R}_{0}(t)$ will be secondary infections belonging to a specific age group:

1 $$R_{0}^{G} (t) = R_{0} (t) \times p^{G} ,$$

where ${R}_{0}(t)=\sum_{G}{R}_{0}^{G}\left(t\right)$ is satisfied. In our analysis, we will assume that ${p}^{G}=\frac{{P}^{G}}{P}$ is the relative size of group $G$ in the total population.

The number of secondary infections, produced by an infected individual, changes with the susceptible population for each group ${S}^{G}(t)$, i.e., number of people alive in age group *G* with no immunity. This is a common feature of SIR models [10]. At each time *t* we will compute the effective share of the reproduction number for each age group as:

2 $$R^{G} (t) = R_{0}^{G} (t) \times s^{G} (t),$$

where ${s}^{G}(t)=\frac{{S}^{G}\left(t\right)}{{P}^{G}\left(t\right)}$ is the ratio of susceptible population overpopulation alive in group $G$ at period $t$. Each infected person at time $t$ will infect ${R}^{G}\left(t\right)$ people from the susceptible population in group $G$. We can define the probability of one infected person infecting a person in group *G* as $\frac{{R}^{G}\left(t\right)}{{S}^{G}\left(t\right)}$; this can be interpreted as the proportion of the susceptible population that will become infected by one infected person. If secondary cases produced by infected people are independent, we can compute the proportion of the susceptible population in group $G$ that will be infected by any person as:

3 $$i^{G} (t) = 1 - \left[ {1 - \frac{{R^{G} (t)}}{{S^{G} (t)}}} \right]^{I\left( t \right)} .$$

In probabilistic terms, the proportion ${i}^{G}\left(t\right)$ can be understood as the probability that a ball is extracted from an urn at least once after $I(t)$ independent extractions with replacement, where $\frac{{R}^{G}\left(t\right)}{{S}^{G}\left(t\right)}$ is the probability of being extracted at a single extraction and ${\left[1-\frac{{R}^{G}\left(t\right)}{{S}^{G}\left(t\right)}\right]}^{I(t)}$ is the probability of a ball not being extracted at any extraction. Finally, the number of new infections in age group $G$ in the next period will be computed as:

4 $$I^{G} (t + 1) = i^{G} (t) \times S^{G} (t).$$

The total new infections in the whole population will be the sum of infections across age groups, i.e., $I\left(t+1\right)=\sum_{G}{I}^{G}\left(t+1\right)$.

Infected individuals at time $t$ will end up having severe symptoms in the next period $t+1$, with a probability ${\mathrm{sev}}^{G}$, or mild or no symptoms, with a probability $1-{\mathrm{sev}}^{G}$, for each group $G$. New individuals with mild or no symptoms will be computed as:

5 $$M^{G} (t + 1) = I^{G} (t) \times (1 - {\text{sev}}^{G} ).$$

Severe cases in period $t+1$ will be:

6 $${\text{SEV}}^{G} (t + 1) = I^{G} (t) \times {\text{sev}}^{G} .$$

Notice that the probability of severe symptoms is assumed to be time independent. On the contrary, the probability of death conditional on developing severe symptoms is included as a time-varying parameter because it will increase if the healthcare system is overwhelmed. Those with severe symptoms in $t+1$ could die with probability $\mathrm{d}{s}^{G}(t+1)$ or survive with $1-\mathrm{d}{s}^{G}(t+1)$ probability. Similarly, those with mild symptoms in $t+1$ could die with probability $\mathrm{d}{m}^{G}(t+1)$. Therefore, total deaths from cases of COVID-19, in period $t+1$, will be computed as:

7 $${\text{DC}}^{G} (t + 1) = {\text{SEV}}^{G} (t + 1) \times {\text{d}}s^{G} (t + 1) + M^{G} (t + 1) \times {\text{d}}m^{G} (t + 1).$$

${\mathrm{DC}}^{G}\left(t+1\right)$ is assumed to include all-cause death cases for COVID-19 patients (i.e., including background and COVID-19 specific mortality) so as to be consistent with the evidence available [11].^2^

It will be considered that all severe cases will require hospitalisation. Consequently, the healthcare system will be overwhelmed at time $t+1$ if total severe cases, $\mathrm{SEV}\left(t+1\right)=\sum_{G}{\mathrm{SEV}}^{G}\left(t+1\right)$, are above hospital capacity $C$. Formally, $\mathrm{d}{s}^{G}(t+1)$ is determined as:

8 $${\text{d}}s^{G} (t + 1) = \left\{ \begin{gathered} \hfill {\text{d}}s^{G} \;\;\;\;\;\;\;\;\;\;\;\;\;\;\;\;\;\;\;\;\;\;\;\;\;\;\;{\text{if}}\;C \ge \;{\text{SEV}}\left( {t + 1} \right) \\ \hfill h\left( {t + 1} \right) \times \left( {ds^{G} - 1} \right) + 1\;\;{\text{if}}\;C < \;{\text{SEV}}\left( {t + 1} \right) \\ \end{gathered} \right.,$$

where $\mathrm{d}{s}^{G}$ is mortality probability under normal functioning of the healthcare system, i.e., $C\ge \mathrm{SEV}\left(t+1\right)$, and $h\left(t+1\right)=\frac{C}{\mathrm{SEV}(t+1)}$ is the proportion of severe patients that will receive hospital treatment at $t+1$ under a situation of overwhelming, i.e., $C<\mathrm{SEV}\left(t+1\right)$. In case of overwhelming, death probability is inversely proportional to $h\left(t+1\right)$; ranging from $\mathrm{d}{s}^{G}$ if most of the patients receive treatment, with $h\left(t+1\right)\cong 1$, to 1 if most of the severe patients are not properly treated at the hospital, with $h\left(t+1\right)\cong 0$.

Two alternative scenarios, giving place to two alternative analyses, were considered in the analysis regarding hospital capacity. Under a first assumption, *unlimited hospital capacity*, $C$ will be sufficiently high to meet any number of hospitalisations demanded over the pandemic. In that scenario, the mortality of severe patients will be fixed at $d{s}^{G}$ independently of hospitalisation needs. Under a second assumption, *limited hospital capacity*, $C$ will equal the number of hospital beds available in a country. In this case, mortality will change according to Eq. (8), i.e., increasing when hospitalisation demand is above capacity.

Population alive and not infected at each period could die according to a background mortality probability ${\mathrm{d}b}^{G}$, specific to each age group. Consequently, deaths for non-infected population were computed as:

9 $${\text{DB}}^{G} (t + 1) = \left[ {P^{G} (t) - I^{G} (t)} \right] \times {\text{d}}b^{G} .$$

In the analysis, immunity is achieved either from the previous infection or by vaccination. A vaccine against COVID-19 is assumed to be available at moment $T$ for all those willing to be vaccinated. Two groups will be vaccinated separately. First, a high-risk group $\mathrm{HR}$ will be vaccinated during the period $T$ to ${T}^{\mathrm{HR}}$ in the next proportion ${v}^{G}(t)$:

10 $$v^{G} \left( t \right) = \left\{ {\begin{array}{*{20}c} 0 \\ {\frac{t - T}{{T^{{{\text{HR}}}} - T}}} \\ 1 \\ \end{array} \begin{array}{*{20}l} { {\text{if }} t < T} \hfill \\ { {\text{if }} T \le t \le T^{{{\text{HR}}}} , G \in HR} \hfill \\ { {\text{if }} t > T^{{{\text{HR}}}} } \hfill \\ \end{array} .} \right.$$

A second group with a low-risk profile $\mathrm{LR}$ will be vaccinated during the period ${T}^{\mathrm{HR}}$ to ${T}^{\mathrm{LR}}$ as:

11 $$v^{G} \left( t \right) = \left\{ {\begin{array}{*{20}c} 0 \\ {\frac{{t - T^{{{\text{HR}}}} }}{{T^{{{\text{LR}}}} - T^{{{\text{HR}}}} }}} \\ 1 \\ \end{array} \begin{array}{*{20}l} { {\text{if }} t < T^{{{\text{HR}}}}} \hfill \\ { {\text{if }} T^{{{\text{HR}}}} \le t \le T^{{{\text{LR}}}} , G \in LR} \hfill \\ { {\text{if }} t > T^{{{\text{LR}}}} } \hfill \\ \end{array} .} \right.$$

Only a percentage $wtv$ of the population will be willing to be vaccinated. Out of those vaccinated only a proportion $e$, given by the effectiveness of the vaccine, will become immune. The proportion of the population that will be immune to COVID-19 via vaccination at each time $t$ and for each group $G$ is computed as:

12 $$\omega^{G} (t) = wtv \times v^{G} (t) \times e.$$

The model is run until heard immunity is achieved after vaccination is complete, i.e., when each infected person is producing only one secondary case or less, and new infections approach to zero. Formally, this will occur when^3^:

13 $$R(t) = \sum {_{G} } R^{G} (t) \le 1\;{\text{and}}\;I(t) = \sum {_{G} } I^{G} (t) \approx 0.$$

### Quality-adjusted life years

Total quality-adjusted life years (QALYs) was the sum of QALYs enjoyed by those who die during the pandemic due to COVID-19 and expected QALYs for those who survive. Also, a reduction in QALYs was added for each patient who suffered mild or severe COVID-19 symptoms (assumed similar to QALY losses driven by influenza B) [12]. For each age group, EQ5D population norms weighted lifetime [13]. The expected QALYs enjoyed for those surviving COVID-19 in each age group were computed by weighting national life-tables survival estimates ${S}^{G}\left(Y\right)$ with population norms ${\mathrm{EQ}5D}^{G}\left(Y\right)$ and applying a discount factor to each year period $Y$ since the beginning of the pandemic:

14 $${\text{EXP}}\_{\text{QALY}}^{G} = \sum {_{Y} } \left[ {S^{G} \left( Y \right) \times {\text{EQ}}5{\text{D}}^{G} \left( Y \right) \times \left( {\frac{1}{1 + d}} \right)^{{\left( {Y - 1} \right)}} } \right]$$

A discount rate $d=3.5\%$ was applied in line with UK National Institute for Health and Care Excellence (NICE) recommendations. A monetary value of 30,000 Euros per QALY (within the range of NICE recommendations) was used to compare QALY benefits with other benefits/costs of lockdown across the four European countries considered [14].

### Hospitalisation costs

Each person needing hospitalisation due to COVID-19 was assumed to be in hospital for several days based on available evidence [15]. The number of intensive care unit (ICU) and in normal ward nights depended on patient survival. The number of hospitalisations incurring in costs at each period was capped at $C$ if the assumption of limited hospital capacity was made.

Hospitalisation nights in intensive care and normal ward were valued at UK NHS reference unit costs [16]. Relative inpatient unit cost for France, Italy and Spain estimated by the World Health Organization were used to adjust hospitalisations UK values [17].

### Alternatives

#### Progression of pandemic under no intervention

The course of the pandemic under no intervention will be determined by the value of ${R}_{0}\left(t\right)$ under normal functioning of society at the beginning of the COVID-19 crisis. It is assumed that the basic reproduction number under no intervention will be constant over time. Therefore, only changes in the susceptible population would reduce the speed of producing secondary cases by affecting the effective reproduction number. The analysis assumes ${R}_{0}\left(t\right)=3.32$ (95% confidence interval, CI 2.81–3.82) under no intervention based on a published meta-analysis [18].

#### Lockdown intervention

A lockdown intervention, like those ordered by European Governments in March 2020, will reduce social mobility and increase physical distance by “staying at home” policies, closure of businesses, schools, and banning of non-essential activities. As a result, the basic reproduction number will be reduced during the lockdown. In this analysis, the basic reproduction number is assumed to be reduced to ${R}_{0}\left(t\right)=0.62$ (95% CI 0.37–0.89) under lockdown according to estimates for the UK [19]. Before and after the lockdown period this parameter will be the same as in the case of no intervention.

##### Lockdown benefits

The benefits of the intervention considered in this analysis are the sum of the monetary value of QALYs saved and hospitalisations avoided simulated by running the model for the two alternatives compared (lockdown vs. no intervention).

##### Lockdown costs

A lockdown intervention may cause costs to society in different dimensions like mental health (e.g., depression due to social isolation), physical health (e.g.,: reduced exercise, and delayed healthcare), education (driven by school closures), and reduced economic activity. In this analysis, we will focus on the productivity costs. It is assumed lockdown will have a cost increasing with duration. More specifically, we will assume that there will be a percentage of economic activity that will have to stop as a result of business closures and physical isolation imposed by governments. Productivity losses were set at 22.6% of the gross domestic product (GDP), proportional to the duration of lockdown, following a UK study based on input–output analysis [20]. This figure is very similar to the 20.4% impact on output in the UK economy reported by the UK Office for National Statistics for April 2020.^4^ Other estimates based on expenditure (20%) or on output data (28%) reported elsewhere are more or less close [21]. We also considered a cost related to time to recover normal production after lockdown is lift; three months is assumed to take for the economy to return to normal activity.

To account for uncertainty on productivity costs we used the variability of the 2019–2020 change reported by Eurostat for the first quarter GDP for European countries that ordered a lockdown in March.^5^

#### Soft lockdown

In an alternative analysis, a soft lockdown is assumed to follow three months of full lockdown (i.e., after the period of March–mid-June 2020). In this situation, some businesses and schools may reopen; physical distance could be relaxed as well. Still some controls of the disease are in place; for example, monitoring of outbreaks and related measures, high-risk activities could be limited, and homeworking could be encouraged. In this sense, productivity costs could be lower than in the case of full lockdown but still higher than zero. The basic reproduction number would be higher than under lockdown but lower than with a no-intervention scenario.

### Analysis

Lockdown effectiveness in reducing the basic reproduction number was used to simulate lockdown benefits compared to the situation of no change in four European countries (UK, France, Italy and Spain). The start of lockdown was set at the time it was ordered in each country. In the main analysis, it was assumed that a vaccine would be available in January 2021. Those older than 60 (high-risk group) will be vaccinated during the first 3 months, and those younger than 61 (low-risk group) will be vaccinated during the nine following months. Duration of lockdown and time to availability of vaccine was modified in exploratory analyses. A simulation of the optimal duration of lockdown was performed by comparing benefits (due to QALYs saved and hospitalisation costs avoided) to productivity costs. Finally, an analysis simulated combinations of ${R}_{0}\left(t\right)$ and GDP losses that would make soft lockdown indifferent to both: (a) full lockdown, applied until vaccination is finished, or; (b) no intervention after 3 months of full lockdown. The analysis was run in R [22].

### Parameters, uncertainty and calibration of the model

The model was run using parameters and corresponding uncertainty summarised in the supplementary material (see Table A1 and A2). In the analysis, the population was divided into age groups of one-year width up to 89 years old. People $\ge 90$ years old were grouped together. Age distribution for each country was taken from national statistics. Background mortality and survival probabilities differed by age group according to national life tables. EQ5D population norms also varied by age based on the evidence available for each country [13]. Probability of death, severe symptoms or mild/no symptoms after COVID-19 infection varied by age and was taken from published evidence [11]. The average serial interval (6.3) and 95% confidence interval (5.2–7.6) were taken from a published study [23]. The parameter for willingness to be vaccinated was taken from preferences over coronavirus vaccine described in a European survey [24]. Probability distribution functions for parameters were used based on available evidence, following standard techniques of decision analytic modelling for economic evaluation, and 1000 random simulations were performed for the uncertainty analysis [25, 26].

All the parameters used in the model simulation were taken from real-world evidence published in the literature or assumed as explained in the methodological sections. The only parameter that was calibrated in our analysis is the number of cumulative infections at the beginning of lockdown in March 2020 which was set so that the average simulated cumulative deaths in mid-June 2020 match actual cumulative deaths reported by the European Centre for Disease Prevention and Control for each country. For calibration purposes, the average and 95% confidence interval of the incubation period and onset-to-death time was considered based on previous estimations [27].

## Results

Results under two different assumptions are shown in the next sections: unlimited vs. limited hospital capacity. The main analysis assumed lockdown would last until the vaccination of the population is complete, one year after its availability in January 2021. The reader is informed when those two assumptions, that is time to availability of vaccine and lockdown duration, are modified.

### Calibration of the model

**Fig. 1:**
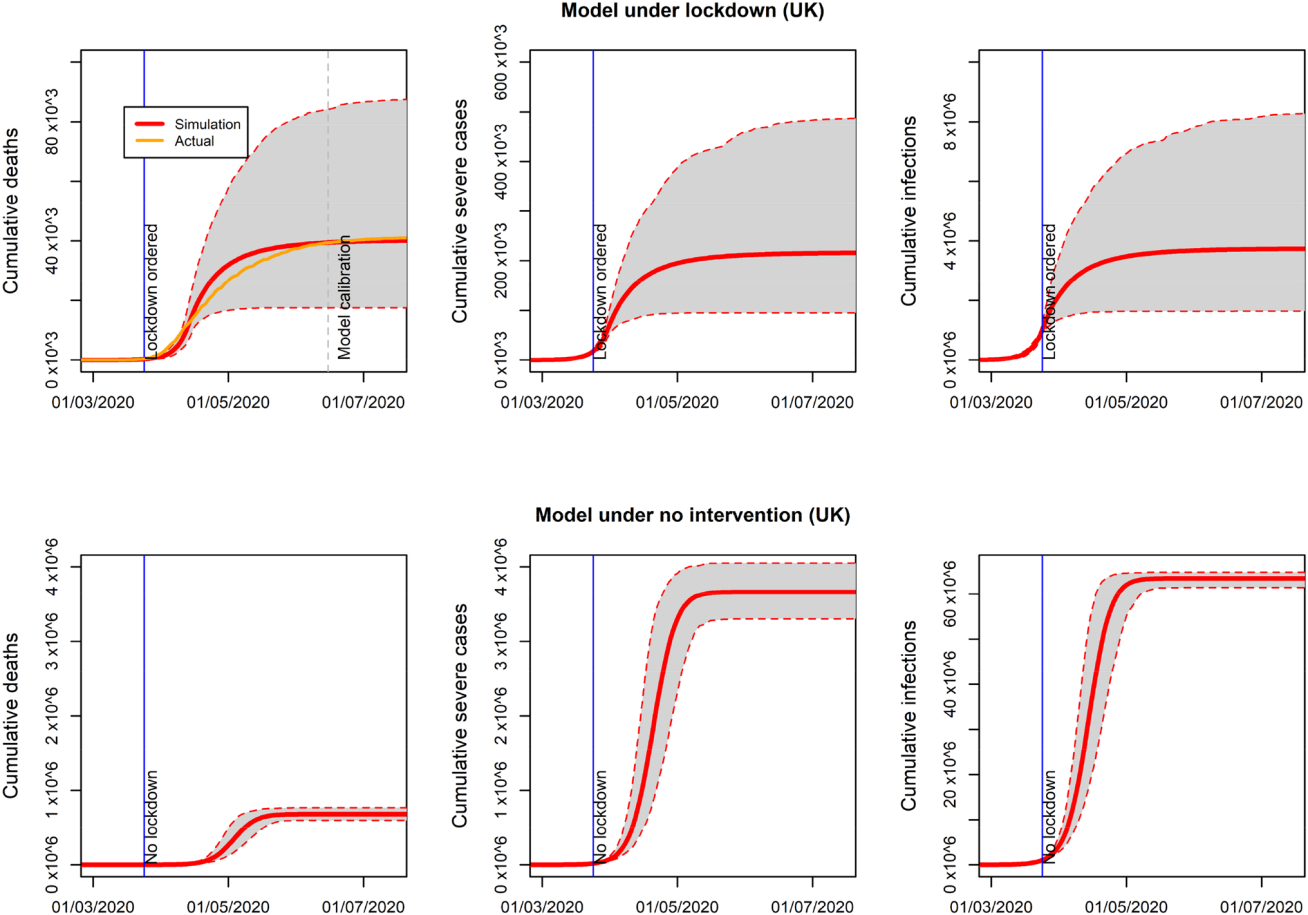
Average and 95% confidence interval of COVID-19 cumulative deaths, hospitalisation demand (severe cases) and infections with/without lockdown. Unlimited hospital capacity assumed (UK). Mean absolute error between daily new death cases simulated and actual deaths reported was 165.5 between March and June

The UK results after calibration of the model under lockdown are compared to the case of no intervention in Fig. 1, when unlimited hospital capacity is assumed, from March to July 2020 (see figures for France, Italy and Spain in supplementary material). The model simulated about 40,000 cumulative deaths on average by 1st July (95% CI 17,576–86,681) under lockdown. Average patients needing hospitalisation would reach 215,171 (95% CI 95,072–483,552) and cumulative infections would be 3,726,904 (95% C, 1,638,203–8,181,999) by 1st of July 2020. Those figures are much higher in the scenario under no intervention where about 95.5% of the population would be infected on average, 680 thousand deaths (95% CI 596,696–768,011) would be expected and about 3.6 million hospitalisations needed (95% CI 3,303,349–4,053,431). The average final size of the pandemic simulated is virtually the same as predicted by the standard continuous-time SIR model with a fully susceptible population when the average reproduction number (3.32) is used [1]. Estimated deaths are consistent with a previous model for the UK accounting for contact patterns between age groups, where about 600 thousands deaths are predicted using a reproduction number of just 3 [2].

**Fig. 2:**
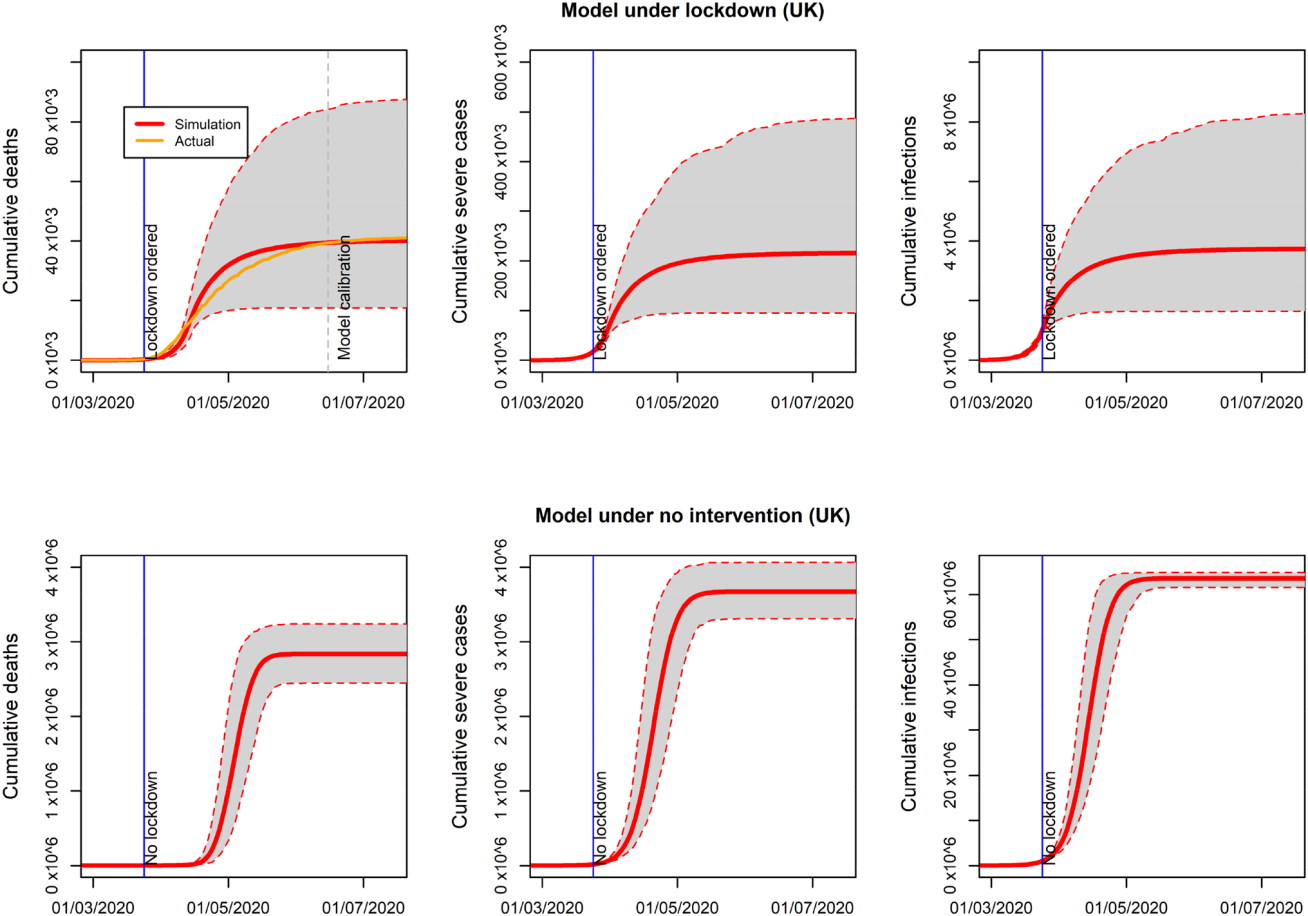
Average and 95% confidence interval of COVID-19 cumulative deaths, hospitalisation demand (severe cases) and infections with/without lockdown. Limited hospital capacity assumed (UK). Mean absolute error between daily new death cases simulated and actual deaths reported was 165.5 between March and June

In Fig. 2, the results under limited hospital capacity are shown. Under lockdown, all the predicted numbers are the same to the case of unlimited hospital capacity (Fig. 1). This is because under lockdown the healthcare system is working below capacity and no additional COVID-19 mortality is predicted by the model. However, under no intervention the number of deaths is a big proportion of hospitalisation cases. With an overwhelmed system, many patients getting infected at the same time would not receive hospital treatment and would die.

### Impact of lockdown

The results of the impact of lockdown for each country are shown in Table 1 under both assumptions considered, unlimited vs. limited hospital capacity. In the first scenario, all patients requiring hospitalisation will be hospitalised. However, under limited hospital capacity only a proportion of patients will actually receive treatment at the hospital and, therefore, the number of hospitalisations averted by lockdown will be lower. On the other hand, QALYs saved will be higher if we adjust COVID-19 mortality in case of an overwhelmed system. In Italy, lockdown is predicted to save 6.79 (CI 5.54–8.05) and 36.51 (CI 31.02–41.72) million QALYs on average, in case of unlimited and limited hospital capacity, respectively. The total monetary value of lockdown benefits relative to annual GDP, i.e., the sum of hospitalisation costs avoided and monetary value of QALYs saved, ranges from 8.27% (CI 6.15–10.36%), in the UK, to 14.32% (CI 11.22–17.61%), in Spain, under unlimited hospital capacity. The predicted figures are much increased if limited hospital capacity is assumed, with France (30.40%, CI 23.92–36.54%) and Spain (70.10%, CI 59.71–80.16%) with the lowest and highest relative benefit, respectively. Hospital capacity is also related to the benefits expected from lockdown across countries. For example, France and Italy would avoid a similar number of severe cases by use of lockdown, however, QALYs saved would be higher for the latter due to a smaller number of hospital beds available and hence a higher risk of an overwhelmed system.

**Table 1:** Impact and benefit of lockdown vs. no intervention

	UK			Spain			Italy			France		
	Mean	95% CI		Mean	95% CI		Mean	95% CI		Mean	95% CI	
Unlimited hospital capacity												
Deaths avoided (10^6^)	0.56	0.49	0.64	0.44	0.39	0.50	0.66	0.58	0.75	0.66	0.57	0.74
Hospitalisation cases avoided (10^6^)	3.45	3.04	3.83	2.65	2.36	2.96	3.76	3.34	4.18	3.76	3.35	4.19
Actual hospitalisations avoided (10^6^)	3.45	3.04	3.83	2.65	2.36	2.96	3.76	3.34	4.18	3.76	3.35	4.19
QALYs saved (10^6^)	5.14	3.84	6.30	4.48	3.57	5.40	6.79	5.54	8.05	6.18	4.86	7.50
QALYs monetary benefits (a) (€10^12^)	0.15	0.12	0.19	0.13	0.11	0.16	0.20	0.17	0.24	0.19	0.15	0.22
Hospitalization costs saved (b) (€10^12^)	0.05	0.04	0.07	0.03	0.02	0.04	0.05	0.04	0.06	0.05	0.04	0.07
Total benefit (a + b) (€10^12^)	0.20	0.15	0.26	0.17	0.13	0.21	0.25	0.20	0.31	0.24	0.19	0.30
GDP (€10^12^)	2.48	2.48	2.48	1.17	1.17	1.17	1.92	1.92	1.92	2.56	2.56	2.56
Benefit as % of GDP	8.27	6.15	10.36	14.32	11.22	17.61	13.19	10.56	15.95	9.34	7.25	11.55
Limited hospital capacity												
Deaths avoided (10^6^)	2.62	2.27	3.00	2.06	1.79	2.34	2.79	2.42	3.20	2.13	1.74	2.54
Hospitalisation cases avoided (10^6^)	3.46	3.04	3.85	2.66	2.37	2.97	3.77	3.36	4.20	3.77	3.36	4.20
Actual hospitalisations avoided (10^6^)	0.82	0.51	1.01	0.59	0.39	0.73	0.99	0.75	1.18	1.85	1.57	2.10
QALYs saved (10^6^)	30.46	25.83	34.74	27.01	23.08	30.80	36.51	31.02	41.72	25.04	19.74	29.97
QALYs monetary benefits (a) (€10^12^)	0.91	0.77	1.04	0.81	0.69	0.92	1.10	0.93	1.25	0.75	0.59	0.90
Hospitalization costs saved (b) (€10^12^)	0.01	0.01	0.02	0.01	0.00	0.01	0.01	0.01	0.02	0.03	0.02	0.03
Total benefit (a + b) (€10^12^)	0.93	0.78	1.06	0.82	0.70	0.93	1.11	0.94	1.27	0.78	0.61	0.93
GDP (€10^12^)	2.48	2.48	2.48	1.17	1.17	1.17	1.92	1.92	1.92	2.56	2.56	2.56
Benefit as % of GDP	37.39	31.56	42.80	70.10	59.71	80.16	57.78	48.98	66.22	30.40	23.92	36.54

Note 1. Alternative assumptions about hospital capacity used

Note 2. Deaths avoided include COVID-19 and background deaths reduction under lockdown vs. no intervention

Note 3. QALYs saved is the difference in total QALYs expected to be lived by the population under the two scenarios compared

Note 4. A quality-adjusted life year (QALY) is given a monetary value of €30,000

Note 5. An exchange rate of 1.12€/£ has been used to convert monetary quantities in the UK

Monetary value of QALYs saved is more important than the monetary value of hospitalisations costs averted in all countries. Especially, under the assumption of limited hospital capacity the average hospitalisation costs avoided are less than 3.8% of total benefits in all the countries.

### Lockdown duration and availability of vaccine

**Fig. 3:**
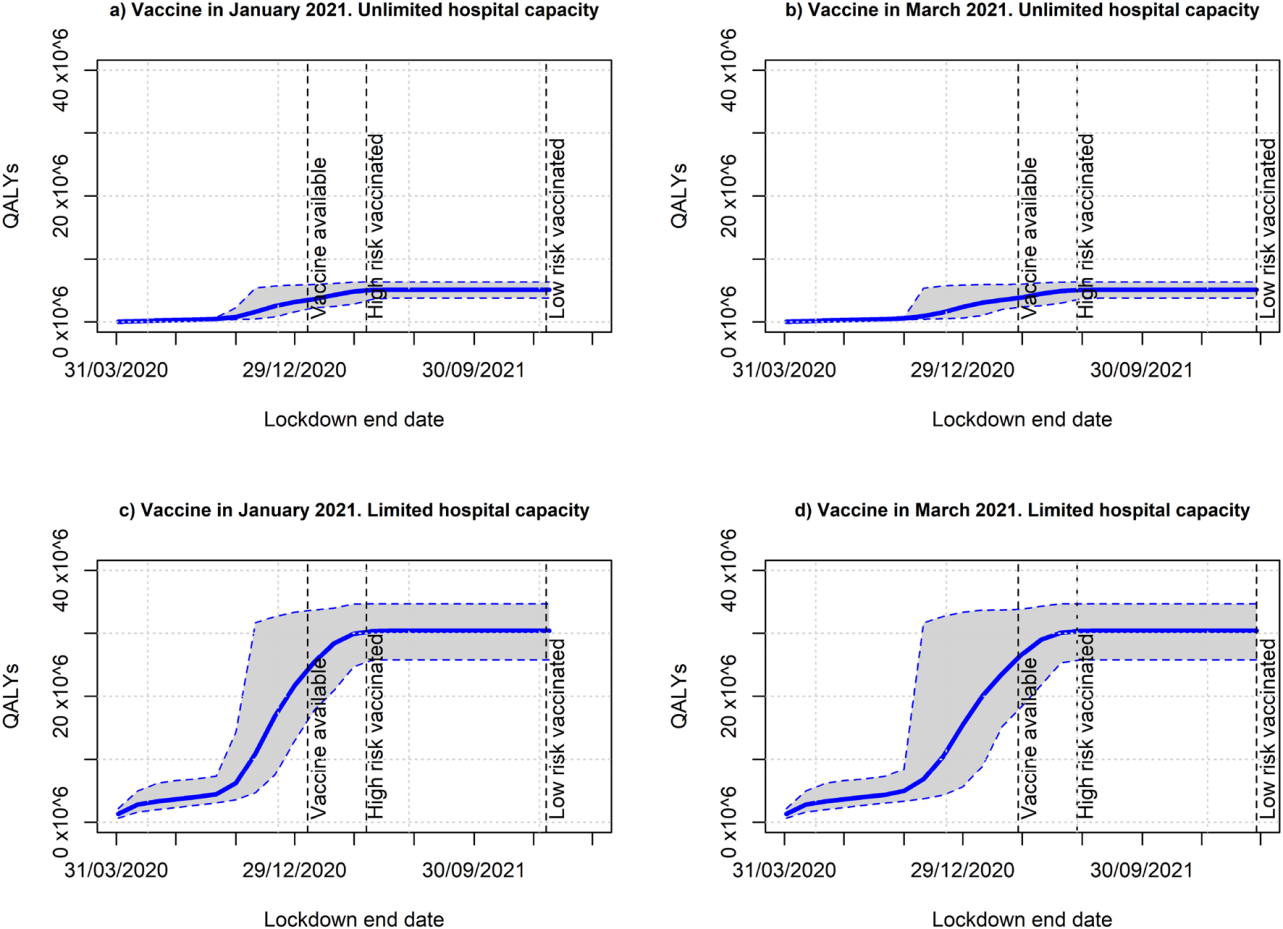
Average and 95% confidence interval of QALYs saved by lockdown duration and time to an available vaccine. Unlimited and limited hospital capacity considered (UK)

Figure 3a and b show QALYs saved assuming different dates of lockdown end for the UK under the assumption of unlimited hospital capacity. Figure 3c and d show the same analysis assuming limited hospital capacity. In addition to the main analysis scenario where a vaccine is available from January 2021, we also consider the alternative scenario where it is available two months later in March 2021.

The number of QALYs saved is increasing with the duration of lockdown drawing an S-shape curve. The effect of lockdown duration on QALYs saved changes over time. Extending lockdown just for a few months, after its ordering in March, will increase QALYs saved moderately. The justification for this result is that a short period of lockdown followed by no intervention will not prevent a second wave to happen before the availability of a vaccine. It is only when the lockdown end date is close to the availability of vaccines that QALYs saved increase substantially with duration. Finally, extending lockdown beyond the moment when the high-risk group is vaccinated will bring almost no benefits. This S-shape pattern is found for the two assumptions on hospital capacity and independently on the time of availability of the vaccine, i.e., to achieve substantial benefits lockdown will need to last until complete vaccination of the high-risk group.

The results for France, Italy and Spain, regarding the shape of the effect of lockdown duration on QALYs saved, are very similar to the abovementioned results for the UK (see supplementary material).

### Optimal lockdown duration

**Fig. 4:**
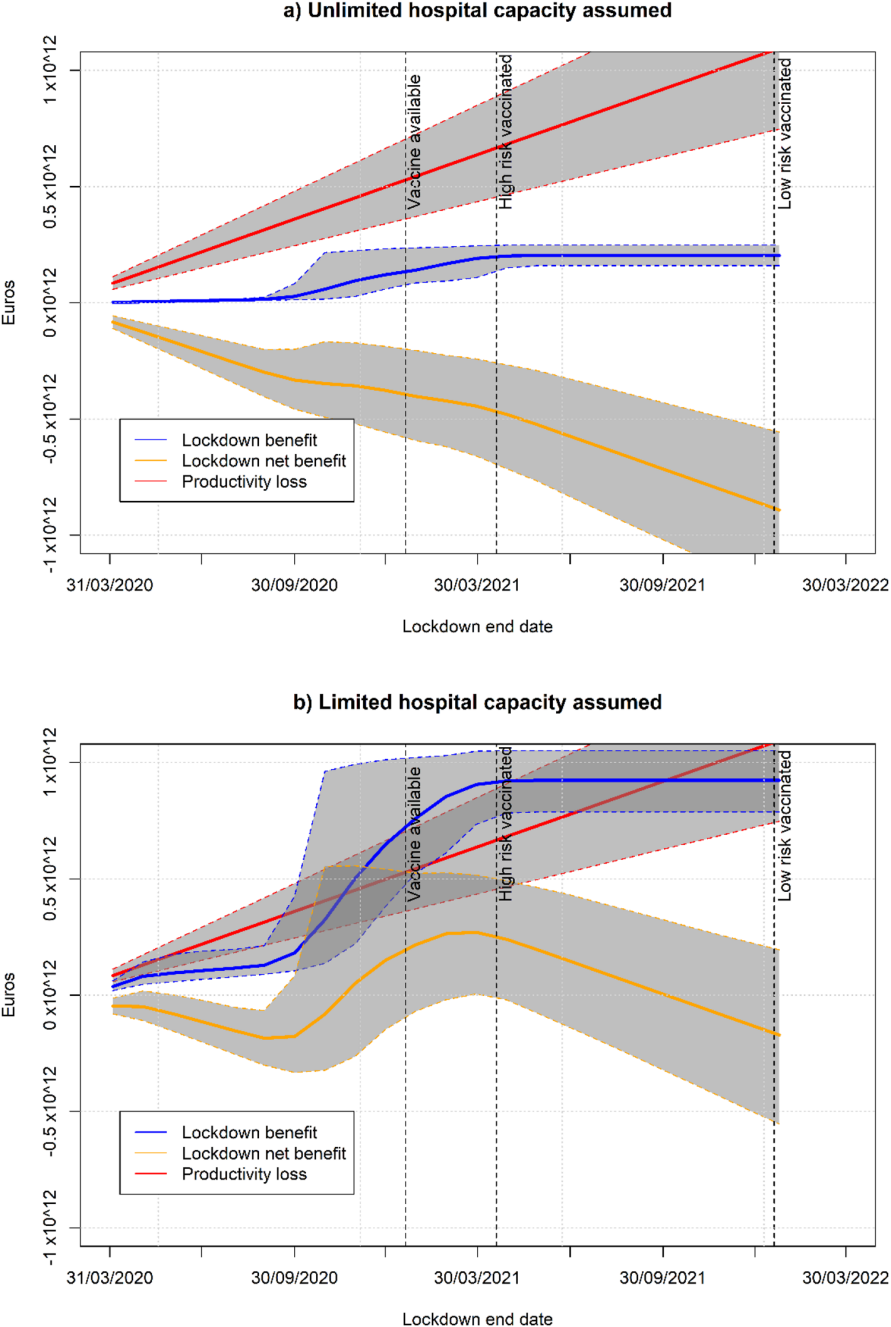
Average and 95% confidence interval of monetary benefit (value of QALYs plus hospitalisations costs saved), productivity losses and net benefit by lockdown duration. Unlimited and limited hospital capacity considered (UK)

Figure 4a and b compare lockdown benefits and costs for the UK depending on the intervention duration for the two alternative hospital capacity assumptions. The analysis assumes a vaccine will be available in January 2021. Benefits are valued in euros and include monetary value of QALYs saved plus hospitalisation savings under lockdown. Costs include productivity losses during the lockdown and a period of economic recovery. The optimal lockdown duration should maximise net benefit, i.e., benefits minus costs. Under unlimited hospital capacity, the average and 95% CI of the net benefit would be negative at any duration of lockdown. Furthermore, the average net benefit will decrease with lockdown duration in such a way that lockdown benefits will not compensate its costs at any point in time. A simple calculation shows that net benefit will be positive only if productivity losses were below 8% of GDP, approximately, if lockdown is extended up to April 2021, i.e., when the high-risk group were vaccinated.

If the assumption of limited hospital capacity is considered, the average net benefit will be positive for any lockdown finishing between November 2020 and October 2021. However, the maximum average net benefit (€0.27 × 10^12^ about 10.9% of GDP) is achieved if the lockdown is lifted at the end of March 2021. At this point, the likelihood of lockdown being optimal is also the highest according to the 95% confidence interval. Figure 4b also shows that stopping lockdown too early would bring negative net benefit even if lockdown is the optimal strategy when extended for an appropriate time period. In other words, lockdown would bring positive net benefit only if continued for a sufficiently long period of time.

The analysis of optimal lockdown duration for France, Italy and Spain can be found in the supplementary material.

### Switching to soft lockdown

**Fig. 5:**
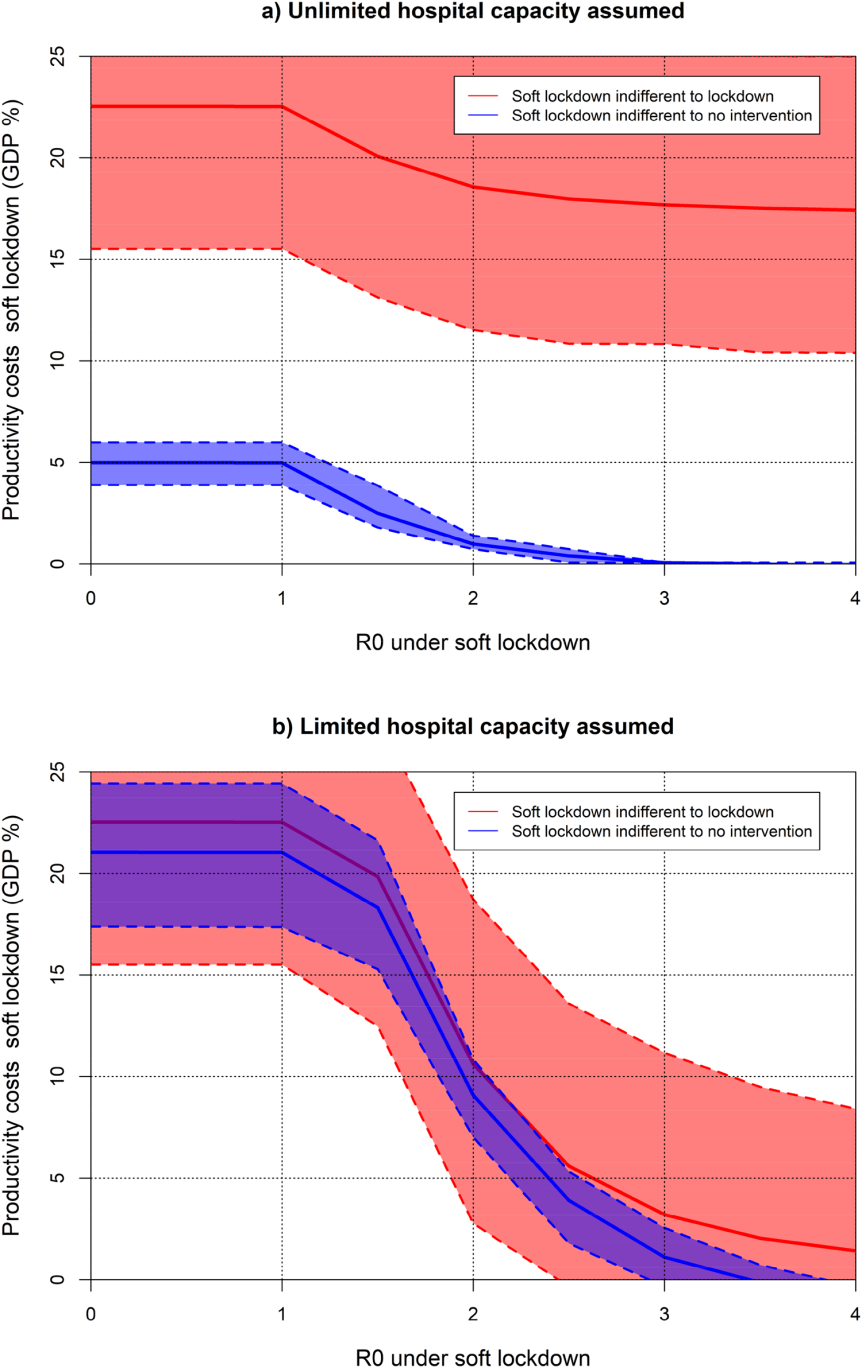
Combinations of ${R}_{0}$ and GDP losses under soft lockdown that would be indifferent to extending lockdown or no intervention from mid-June 2020. Average and 95% confidence interval (UK)

In this section, we consider the impact of switching from full lockdown, from March to mid-June 2020, to soft lockdown, from June until the whole population is vaccinated, assumed in January 2022. The easing of lockdown was compared to continued lockdown or no intervention in any form for the same period. The UK Fig. 5a and b show the characteristics (basic reproduction number and productivity costs) of soft lockdown to be indifferent (same net benefit) to full lockdown or no intervention under unlimited and limited hospital capacity respectively. Since the indifference curve has been computed for each of the 1000 simulations, the average and 95% confidence interval are shown in the Figures accounting for uncertainty. Notice that more efficient strategies are achieved if productivity losses or basic reproduction number are reduced, hence the interesting combinations are those points below the indifference curve that will be more efficient than the comparator (either full lockdown or no intervention).

Under the two hospital capacity assumptions, we find scenarios of soft lockdown that will be the best policy. For example, if the basic reproduction number is maintained at 1 then a soft lockdown with a productivity cost of 3.9% or lower will be the best strategy with a probability higher than 97.5% compared to full lockdown or no intervention and independently of the assumption about hospital capacity. If we focus on the limited hospital capacity assumption, we find more opportunities for the soft lockdown to be the best strategy. Indeed, any soft lockdown with a productivity cost below 15% (12%) will be the best strategy, with a > 97.5% probability, when the reproduction number is maintained at 1 (1.5).

The analysis of soft lockdown for France, Italy and Spain can be found in the supplementary material.

## Discussion

There is a need for making optimal decisions where the benefits of lockdown are consequences of changes in epidemiological parameters. The lack of key information about the effectiveness of interventions requires modelling the link between epidemiological parameters and final outcomes under different plausible scenarios. Furthermore, the characteristics of the target population and evidence on model parameters should be accounted for. While there is available evidence of the effects of public health interventions on epidemiological variables, the final effects on outcomes valued by society, like QALYs saved, is complex. Understanding this complexity can help us to assess the value of policies and to set optimal strategies. The analysis presented shows that the relationship between key variables like time to availability of a vaccine and duration of lockdown is crucial to understand the value of lockdown. For example, if vaccination of high-risk groups is delayed, then lockdown duration should be extended as well to achieve substantial benefits. The analysis of lockdown duration is also important given the increasing costs associated. The results here show that the optimal lockdown end may be at the point when high-risk groups are vaccinated rather than when the whole population is vaccinated. This result can be explained by two aspects: first, the vaccination of the high-risk group will reduce the susceptible population and hence reduce the spread of the virus to those non-vaccinated yet; second, the low-risk group faces a lower risk of death or hospitalisation than the high-risk group. On the other hand, a lockdown stopped too early could have huge costs in terms for QALYs losses due to the plausibility of a second wave.

Results also show the scenarios under which QALYs saved and hospitalisations are avoided may not be enough to justify the huge cost of lockdown in terms of economic activity lost. In this sense, the need for other strategies, soft lockdowns, has been explored as a better alternative to hard lockdown. If these alternatives could alleviate the economic burden while keeping the number of infectious contacts low, they could bring efficiency gains.

The two assumptions considered regarding hospital capacity resulted in substantial differences in the benefits of lockdown. While consequences of a shortage of hospital beds, and hence lack of hospital treatment, on COVID-19 prognosis are unknown, the two scenarios could be considered to understand the plausible range of benefits. Remarkably enough, we found that changing the assumption about hospital capacity will affect the results more than considering statistical uncertainty in the model parameters.

The model allowed us to introduce relevant aspects for economic evaluation and was also consistent with standard epidemiological models. For example, the final size of the pandemic predicted is very similar to predictions of the standard continuous-time SIR model [1]. The model is also flexible in adapting to the characteristics of different countries like age distribution of the population, hospital capacity, hospitalisation costs, mortality rates or country-specific health utilities. For comparability of results across countries, we have considered some common parameter values. For example, QALYs were valued at €30,000 and the same discounting rate was used. However, preferences for allocating resources over time and between health and other dimensions may differ between countries. This figure is nonetheless within the range of annual GDPs per capital in the four countries considered. It is also within the limits of the threshold used in healthcare decisions advised by NICE in UK (between £20,000 and £30,000) [14].

The present analysis is related to previous studies aimed at evaluating non-pharmaceutical interventions based on dynamic epidemiological models. For example, Shlomai et al. [28] performed a cost-effectiveness analysis of a “testing, tracing, and isolation” approach and a national lockdown strategy; the latter resulting in “tremendous costs to prevent 1 case of death”. While their analysis makes use of real-world evidence (plus assumptions) and correctly acknowledges the need for a dynamic model that is based on transmission rates (and therefore on the basic reproduction number), we can identify some limiting aspects:—duration of interventions is fixed;—time horizon seems too short (200 days); end of the pandemic is not defined (e.g., via vaccination available), and; hospital beds capacity of the national health system is ignored. In a rapid benefit–cost analysis, Linda et al. [29] accounted for the possibility of an overwhelmed health care system and made use of the concept of the value of statistical life (VSL) for the evaluation of fixed-duration social distancing [29]. Even though the VSL is a valid framework to perform economic evaluation, its use in the case of COVID-19 interventions should be discussed given the heterogeneity in death rates across age groups. Up to now, the studies performing economic evaluations of COVID-19 non-pharmaceutical interventions based on dynamic epidemiological models are limited and do not account for the effect of lockdown duration [30]. On the contrary, studies in the epidemiological literature have shown the importance of including the duration of non-pharmaceutical interventions as a relevant model parameter but they fail to value the costs and benefits of lockdowns and social distance measures [3].

This study has some limitations and does not consider other important dimensions such as mental health, education or domestic violence. In addition, the consequences of an overwhelmed system on non-COVID-19 patients have been omitted from the analysis. It would also be difficult to account for the impact of an uncontrolled pandemic, e.g., 90% of the population getting infected in a short period of time, on economic activity. All these aspects should be considered in the decision-making process, in addition to the implications for democratic and ethical principles. Finally, our analysis assumes the basic reproduction number under no intervention is constant over time, consistently with previous work [3]. Even though we allowed for the effective reproduction number to change over time according to changes in the susceptible population, future research could explore behaviour-driven changes.

## Supplementary Information

Below is the link to the electronic supplementary material.

Supplementary file 1 (DOCX 4042 KB)

## Footnotes

1 See link: https://www.lshtm.ac.uk/research/centres/global-health-economics-centre/covid-19.

2 In this analysis, evidence of *all-cause* deaths for severe COVID-19 patients comes from Verity et al. (2020) including background and COVID-19 specific mortality. We will assume that mortality of mild/no symptoms COVID-19 patients will be driven by background mortality according to life tables.

3 Notice, that ${R}^{G}\left(t\right)$ is the number of secondary cases produced in group $G$ by one infected person. Therefore, herd immunity is achieved when the sum $\sum_{G}{R}^{G}\left(t\right)$ is equal to or less than 1.

4 https://www.ons.gov.uk/economy/grossdomesticproductgdp/articles/coronavirusandtheimpactonoutputintheukeconomy/april2020.

5 https://ec.europa.eu/eurostat/documents/portlet_file_entry/2995521/2-14082020-AP-EN.pdf/7f30c3cf-b2c9-98ad-3451-17fed0230b57.
