# Supplementary material for: Reducing the basic reproduction number of COVID-19: a model simulation focused on QALYs, hospitalisation, productivity costs and optimal (soft) lockdown
Source: Eur J Health Econ. 2022 Aug 2;24(4):647–59. doi: 10.1007/s10198-022-01500-7 (PMC9344232; doi:10.1007/s10198-022-01500-7)
Supplement: Supplementary file 1 — (DOCX 4042 KB) [file 10198_2022_1500_MOESM1_ESM.docx]

**Supplementary material: Reducing the basic reproduction number of COVID-19: a model simulation focused on QALYs, hospitalisation, productivity costs and optimal (soft) lockdown**

**Parameters of the model**

Parameters subject to uncertainty are detailed in Table A1 where the used probability distribution function and parameter values are shown. The distribution functions used have been calibrated to meet average estimates and approximate 95% confidence intervals or standard errors reported in published evidence. In the case of probability of COVID-19 death (conditional on severe case), a distribution function was chosen so that, in combination with probability of severe case, approximate the distribution of COVID-19 mortality (conditional on infection) for each age group.^1^ Willingness to be vaccinated was based on an European survey were individual indicated whether they would be willing to be vaccinated against the coronavirus. ^2^ Observations for yes/no answers were used as beta and alfa parameters in a Beta distribution after assuming that 50% of unsure individuals will be vaccinated. Productivity losses were approximated with a normal distribution where the mean was equal to simulation estimates for the UK. ^3^ In order to calibrate the standard deviation we used the variability of the 2019-2020 change reported by Eurostat for the first quarter GDP for European countries that ordered a lockdown in March (<https://ec.europa.eu/eurostat/documents/portlet_file_entry/2995521/2-14082020-AP-EN.pdf/7f30c3cf-b2c9-98ad-3451-17fed0230b57>). The proportional annual change in GDP was computed using the number of days in lockdown reported elsewhere.^4^ The standard error of the proportional change in 10 countries (Belgium, Denmark, Germany, Spain, France, Italy, Austria, UK, Norway and Switzerland) were used in the analysis.

Parameters specific to each country included in this study are reported in Table A2.

| Table A1. Parameters and probability distribution | | | | |
| --- | --- | --- | --- | --- |
|  | **Mean/α** | **SD/β** | **Distribution** | **Source** |
| R0 under no intervention | 170 | 51.20 | Gamma | ^5^ |
| R0 under lockdown | 21.5 | 34.67 | Gamma | ^6^ |
| Serial interval (days) | 2.26 | 0.3587 | Gamma | ^7^ |
| QALY lost due to mild covid-19 symptoms | 0.00336 | 0.0095 | Normal | ^8^ |
| QALY lost due to severe covid-19 symptoms | 0.01501 | 0.0095 | Normal | ^8^ |
| Hospital length of stay (non-survivor) | 24 | 3.2 | Gamma | ^9^ |
| ICU length of stay (non-survivor) | 15 | 1.875 | Gamma | ^9^ |
| Hospital length of stay (survivor) | 15 | 2.142 | Gamma | ^9^ |
| ICU length of stay (survivor) | 62 | 5.17 | Gamma | ^9^ |
| Productivity loss | 0.226 | 0.03918 | Normal | ^3^ |
| Willingness to be vaccinated | 6388 | 1276 | Beta | ^2^ |
| Infection to death time |  |  |  | ^10^ |
| Incubation period | Log(5) | 0.1 | Lognormal |  |
| Onset to death time | Log(14.5) | 0.08 | Lognormal |  |
| Probability of COVID-19 severe case |  |  |  | ^1^ |
| Age 0-9 | 0.05 | 3105.54 | Beta |  |
| Age 10-19 | 7.00 | 17149.86 | Beta |  |
| Age 20-29 | 7.00 | 666.08 | Beta |  |
| Age 30-39 | 7.00 | 197.08 | Beta |  |
| Age 40-49 | 9.80 | 220.79 | Beta |  |
| Age 50-59 | 6.40 | 72.03 | Beta |  |
| Age 60-69 | 6.20 | 46.34 | Beta |  |
| Age 70-79 | 5.80 | 29.14 | Beta |  |
| Age >79 | 5.60 | 24.83 | Beta |  |
| Probability of COVID-19 death (conditional on severe case) |  |  |  | ^1^ |
| Age 0-9 | 1.00 | 0.00 | Beta |  |
| Age 10-19 | 0.08 | 0.39 | Beta |  |
| Age 20-29 | 3.40 | 111.03 | Beta |  |
| Age 30-39 | 18.00 | 713.52 | Beta |  |
| Age 40-49 | 17.00 | 431.76 | Beta |  |
| Age 50-59 | 35.00 | 445.00 | Beta |  |
| Age 60-69 | 400.00 | 2045.60 | Beta |  |
| Age 70-79 | 1000.00 | 2878.50 | Beta |  |
| Age >79 | 1000.00 | 1358.97 | Beta |  |
| α and β are the parameters of Gamma or Beta distributions. SD – standard deviation | | | | |

| Table A2. Parameters specific to each country | | | | | |
| --- | --- | --- | --- | --- | --- |
|  | **UK** | **France** | **Italy** | **Spain** | **Source** |
| Population (million) | 66.4 | 67.1 | 60.4 | 47 | Note 1 |
| Age distribution |  |  |  |  | Note 1 |
| Date of lockdown start | 24/03/2020 | 17/03/2020 | 11/03/2020 | 14/03/2020 | ^4^ |
| Background mortality |  |  |  |  | Note 2 |
| Survival probability by age |  |  |  |  | Note 2 |
| EQ5D population norms |  |  |  |  | ^11^ |
| Number of beds per 1000 people | 2.54 | 5.98 | 3.18 | 2.97 | Note 3 |
| ICU night unit cost (Euro) | 1297.82 | 1121.85 | 1032.40 | 1012.16 | ^12^ and ^13^ |
| Hospital night unit cost (Euro) | 482.84 | 417.37 | 384.09 | 376.56 | ^12^ and ^13^ |
| Note 1. UK: <https://www.ons.gov.uk/peoplepopulationandcommunity/populationandmigration/populationestimates/articles/overviewoftheukpopulation/august2019> France: <https://www.insee.fr/en/statistiques/2382597?sommaire=2382613> Italy: <http://demo.istat.it/pop2019/index.html> Spain: [https://www.ine.es/jaxi/Datos.htm?path=/t20/e245/p08/l0/&file=03003.px#!tabs-tabla](https://www.ine.es/jaxi/Datos.htm?path=/t20/e245/p08/l0/&file=03003.px%23!tabs-tabla) | | | | |  |
| Note 2. UK: <https://www.ons.gov.uk/peoplepopulationandcommunity/birthsdeathsandmarriages/lifeexpectancies/datasets/nationallifetablesunitedkingdomreferencetables> France: <https://www.ined.fr/en/everything_about_population/data/france/deaths-causes-mortality/mortality-tables/> Italy: <http://demo.istat.it/tvm2016/index.php?lingua=eng> Spain: <https://www.ine.es/jaxiT3/Tabla.htm?t=27155&L=1> | | | | |  |
| Note 3. [https://ec.europa.eu/eurostat/statistics-explained/index.php/Healthcare_resource_statistics_-_beds#Hospital_beds](https://ec.europa.eu/eurostat/statistics-explained/index.php/Healthcare_resource_statistics_-_beds%23Hospital_beds) | | | | |  |

**Detailed analysis for France**


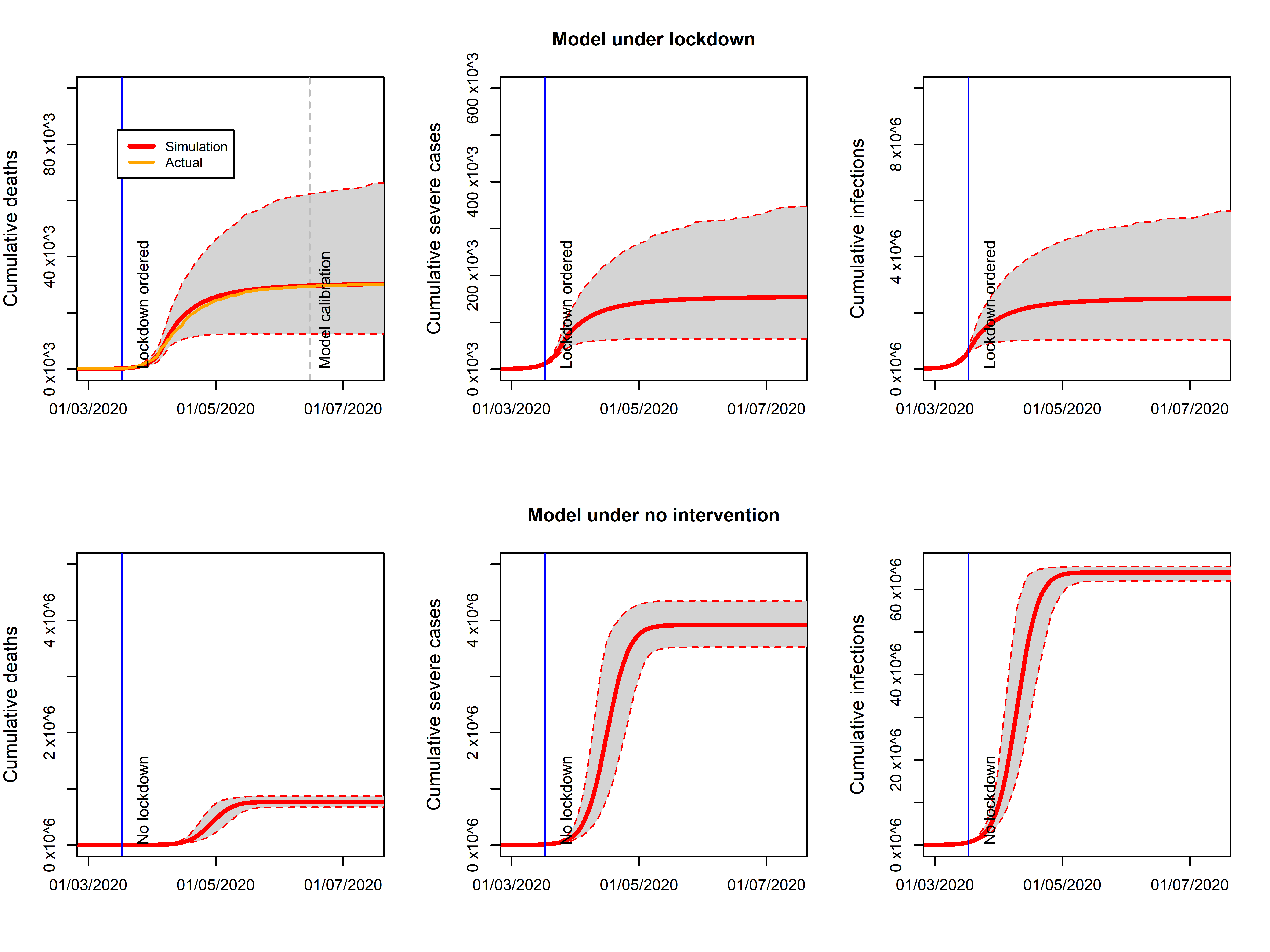


Figure A1. Average and 95% confidence interval of COVID-19 cumulative deaths, hospitalisation demand (severe cases) and infections with/without lockdown. Unlimited hospital capacity assumed (France). Note: Mean absolute error between daily new death cases simulated and actual deaths reported was 83.8 between March and June.


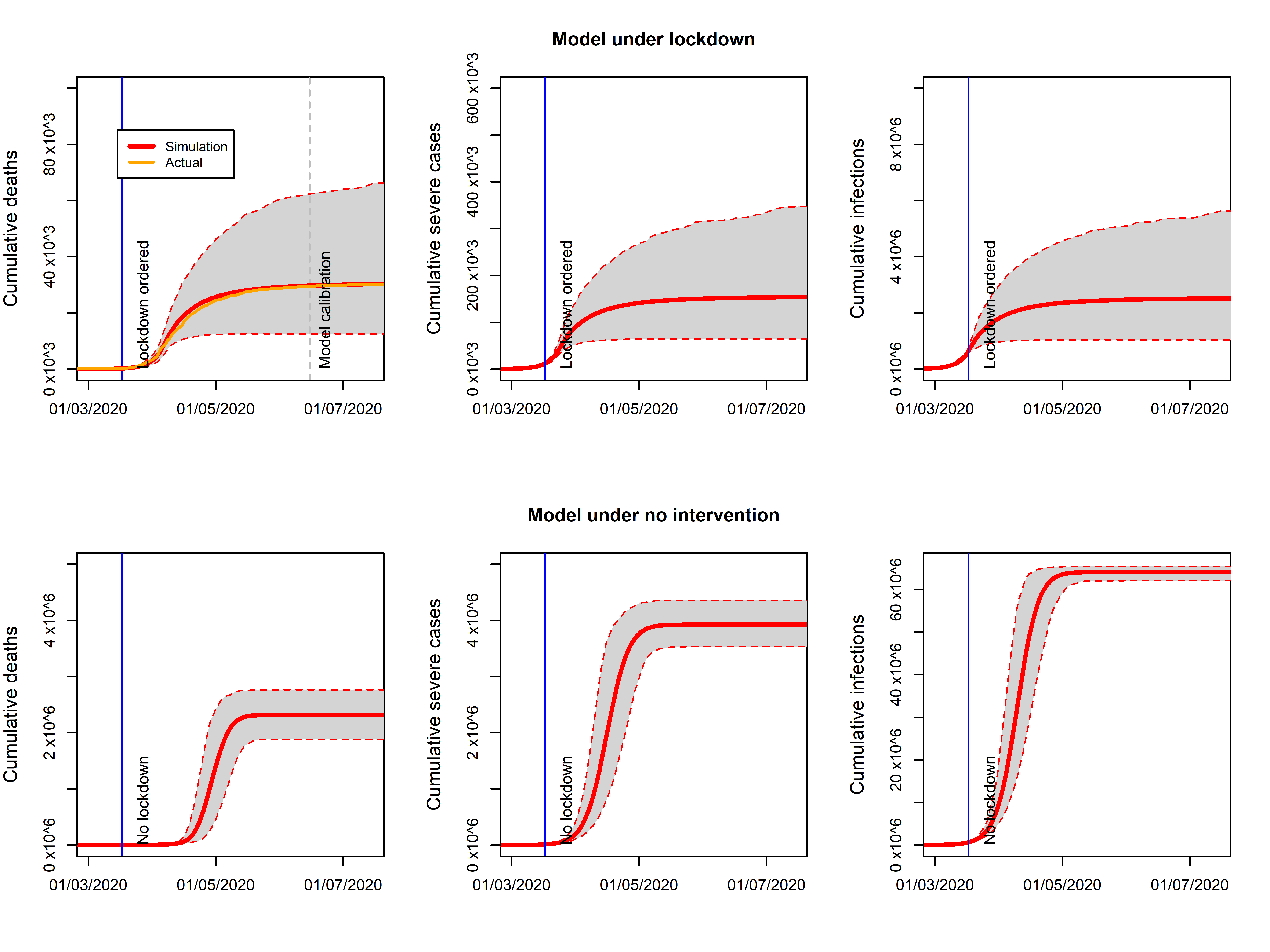


Figure A2. Average and 95% confidence interval of COVID-19 cumulative deaths, hospitalisation demand (severe cases) and infections with/without lockdown. Limited hospital capacity assumed (France). Note: Mean absolute error between daily new death cases simulated and actual deaths reported was 83.8 between March and June.


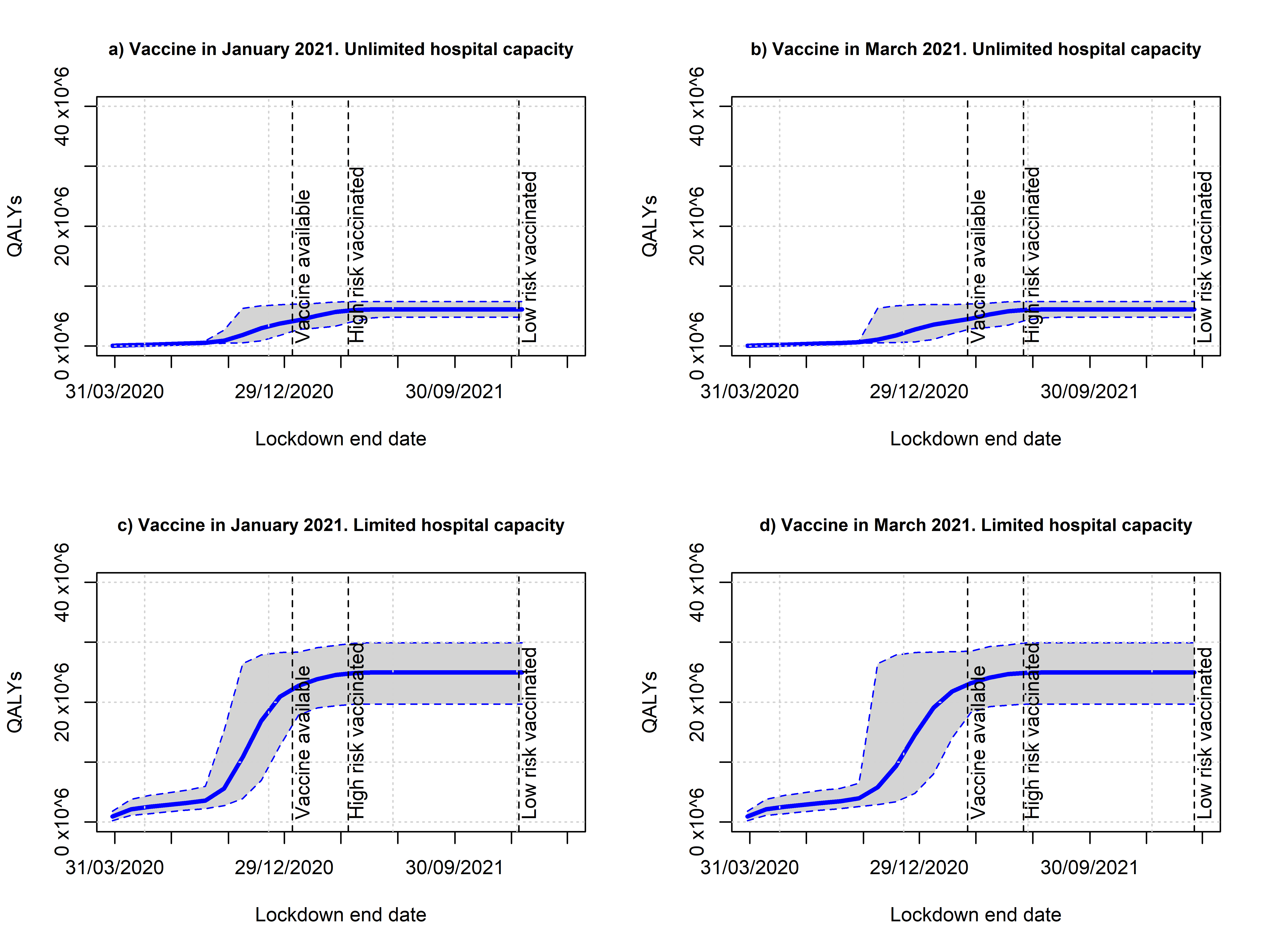
Figure A3. Average and 95% confidence interval of QALYs saved by lockdown duration and time to available vaccine. Unlimited and limited hospital capacity considered (France).


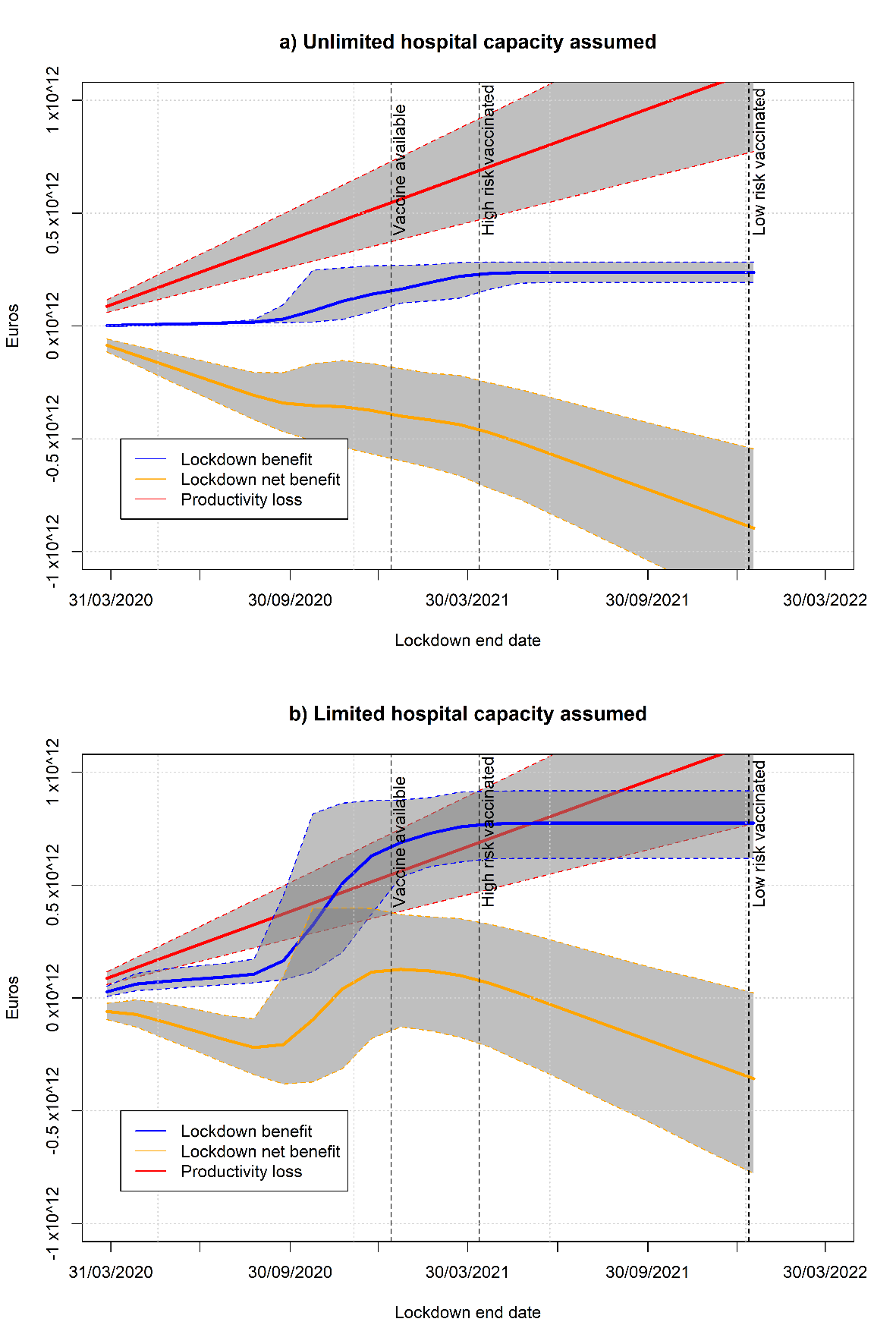


Figure A4. Average and 95% confidence interval of monetary benefit (value of QALYs plus hospitalisations costs saved), productivity losses and net benefit by lockdown duration. Unlimited and limited hospital capacity considered (France)


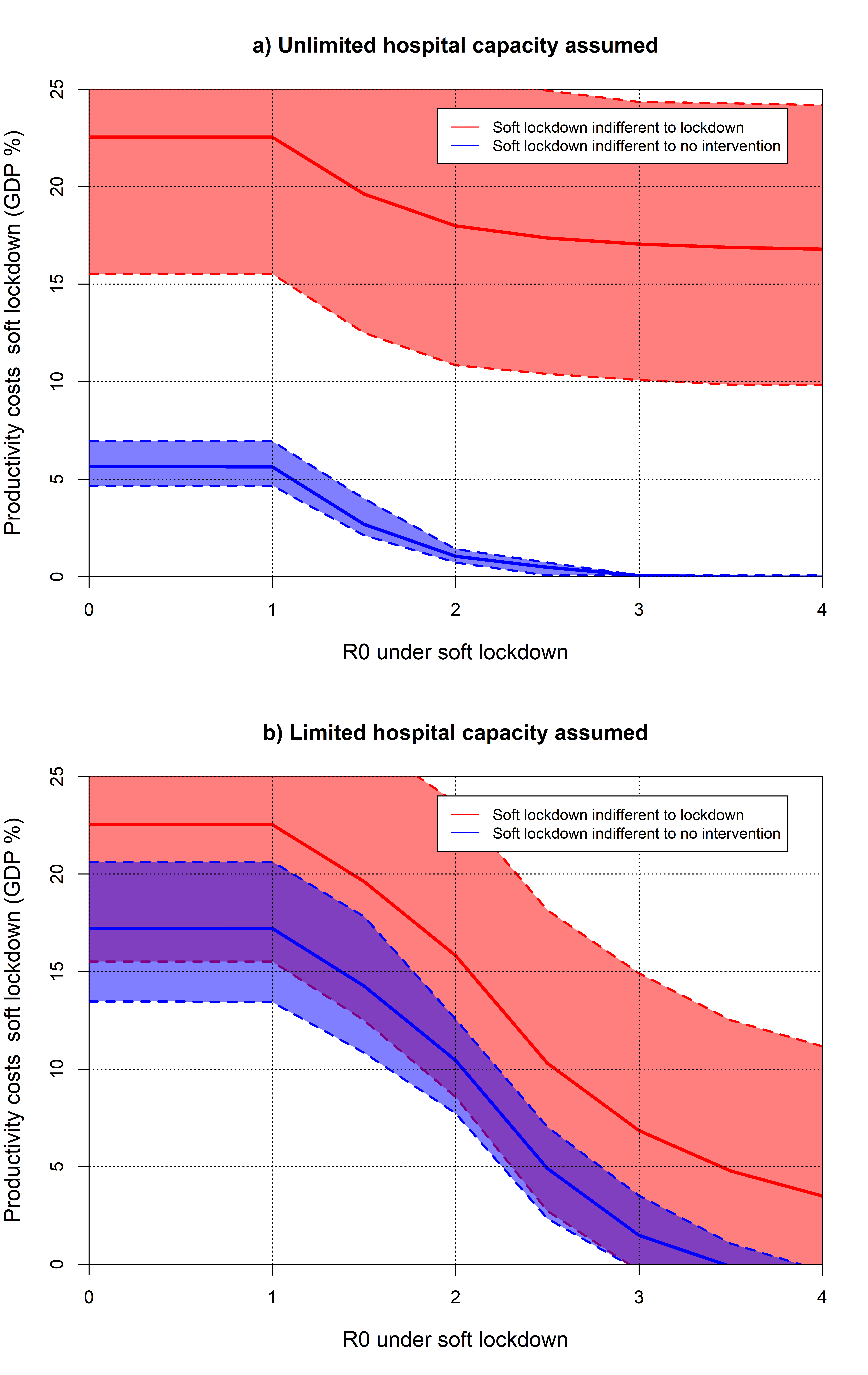


Figure A5. Combinations of $R_{0}$ and GDP losses under soft lockdown that would be indifferent to extending lockdown or no intervention from mid-June 2020. Average and 95% confidence interval (France).

**Detailed analysis for Italy**


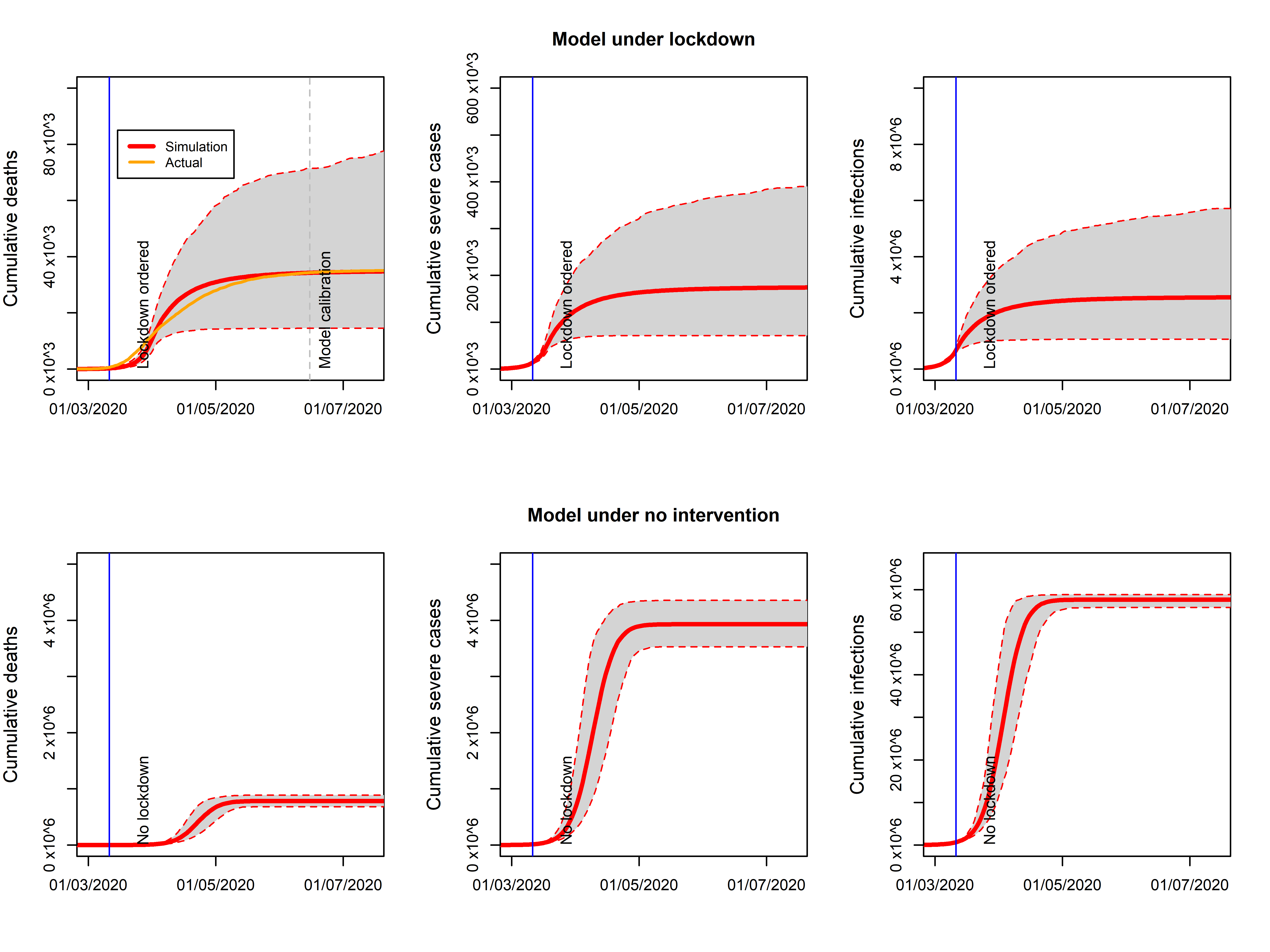


Figure A6. Average and 95% confidence interval of COVID-19 cumulative deaths, hospitalisation demand (severe cases) and infections with/without lockdown. Unlimited hospital capacity assumed (Italy). Note: Mean absolute error between daily new death cases simulated and actual deaths reported was 136.9 between March and June.


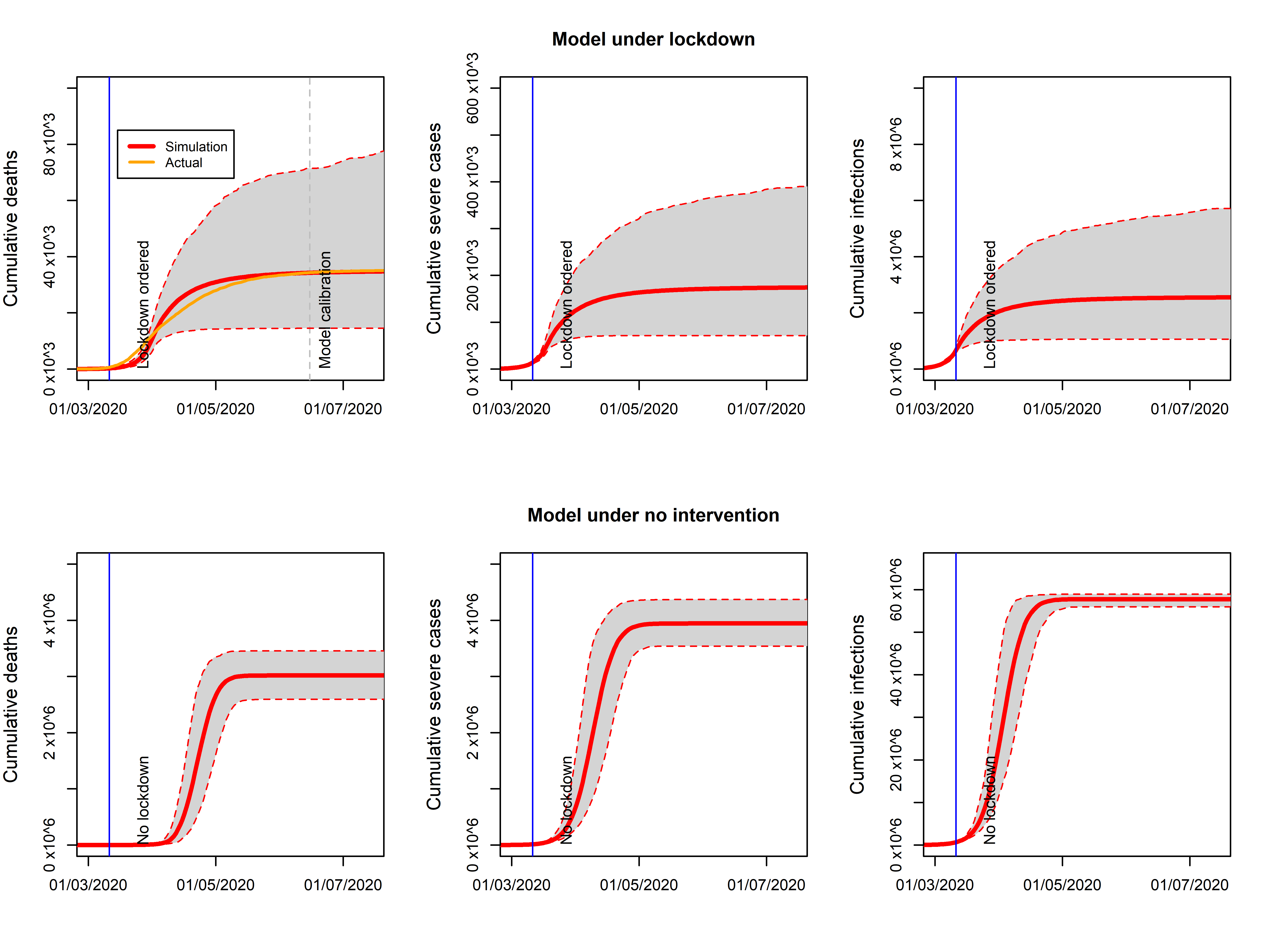


Figure A7. Average and 95% confidence interval of COVID-19 cumulative deaths, hospitalisation demand (severe cases) and infections with/without lockdown. Limited hospital capacity assumed (Italy). Note: Mean absolute error between daily new death cases simulated and actual deaths reported was 136.9 between March and June.


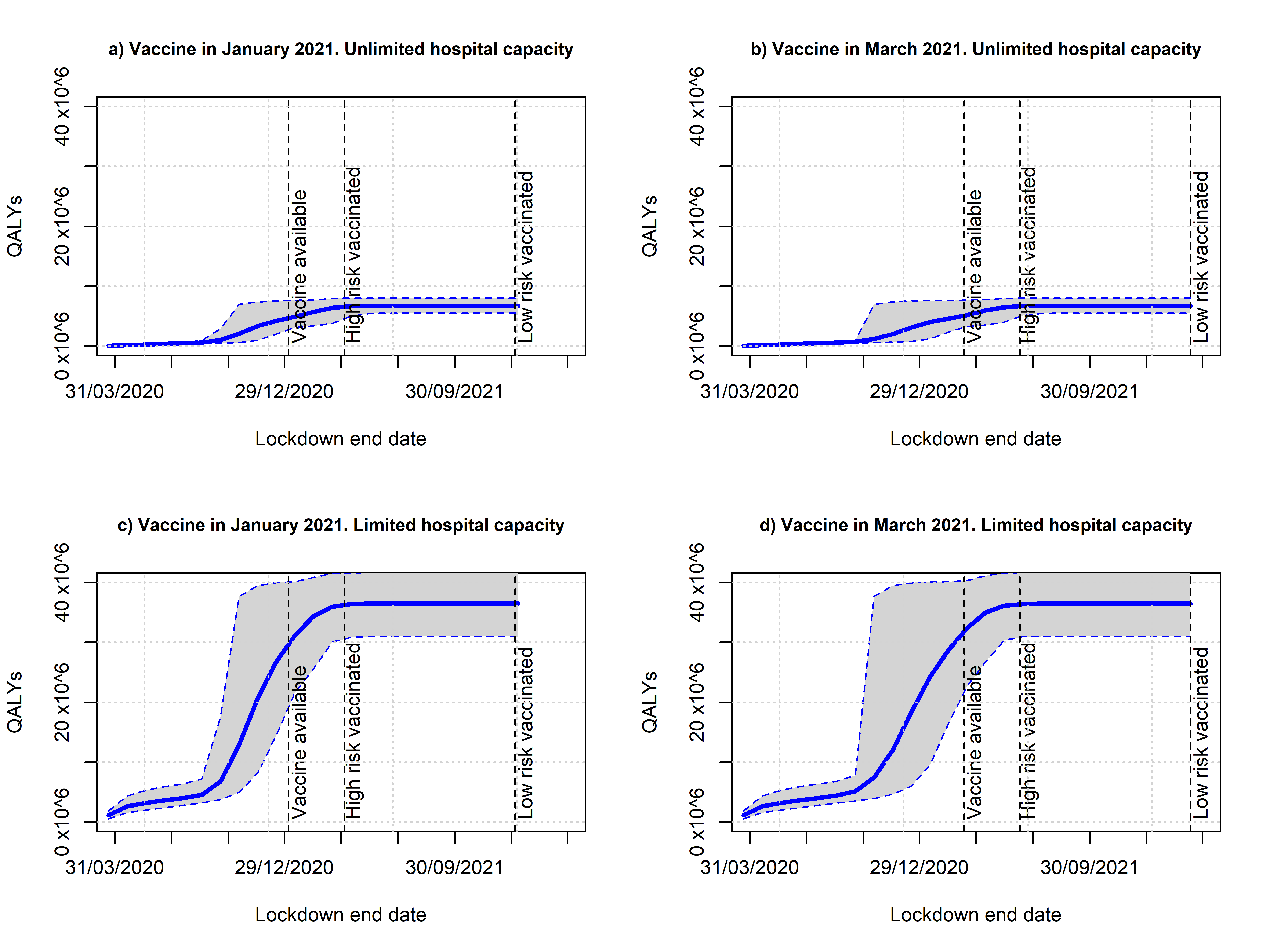
Figure A8. Average and 95% confidence interval of QALYs saved by lockdown duration and time to available vaccine. Unlimited and limited hospital capacity considered (Italy).


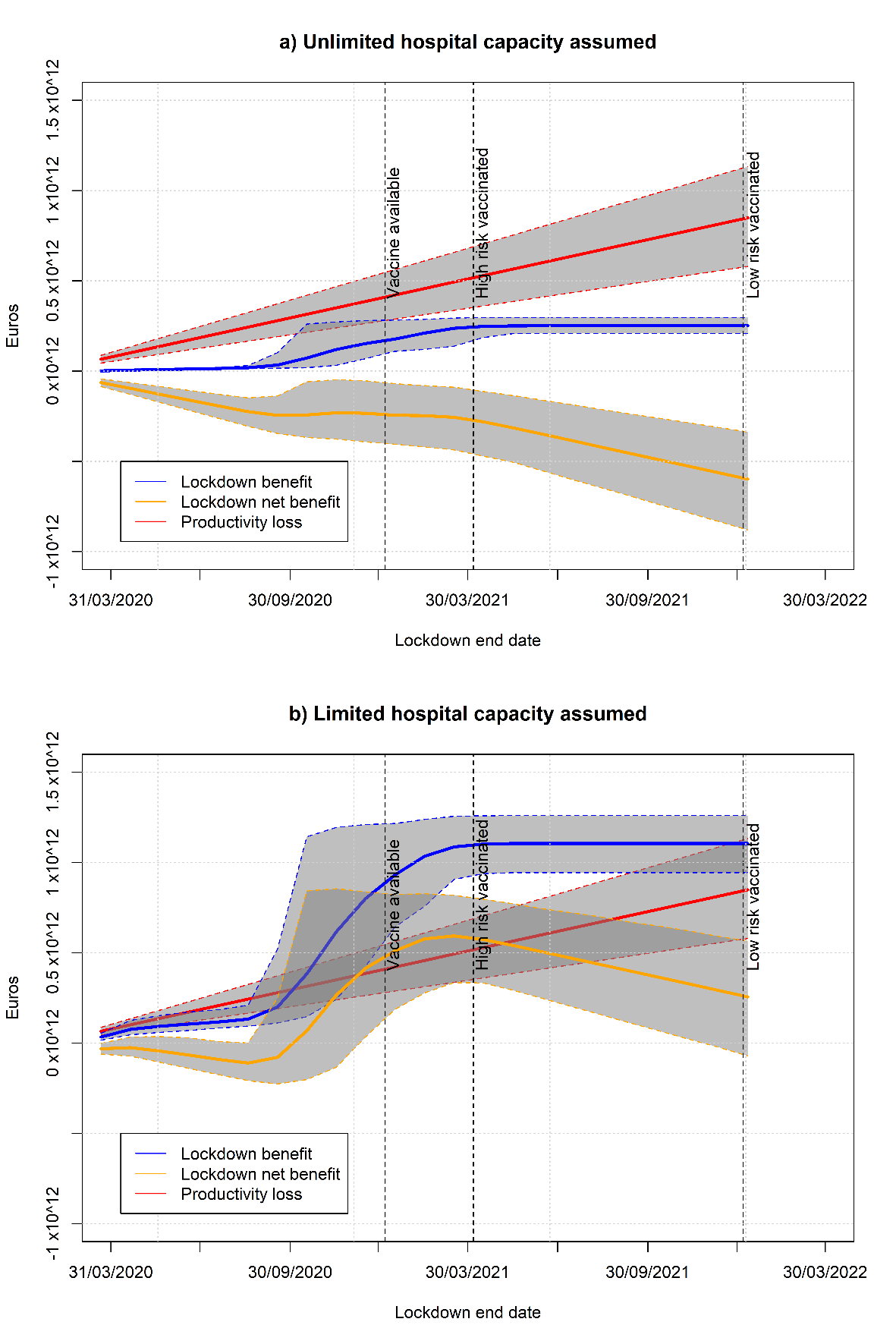


Figure A9. Average and 95% confidence interval of monetary benefit (value of QALYs plus hospitalisations costs saved), productivity losses and net benefit by lockdown duration. Unlimited and limited hospital capacity considered (Italy)


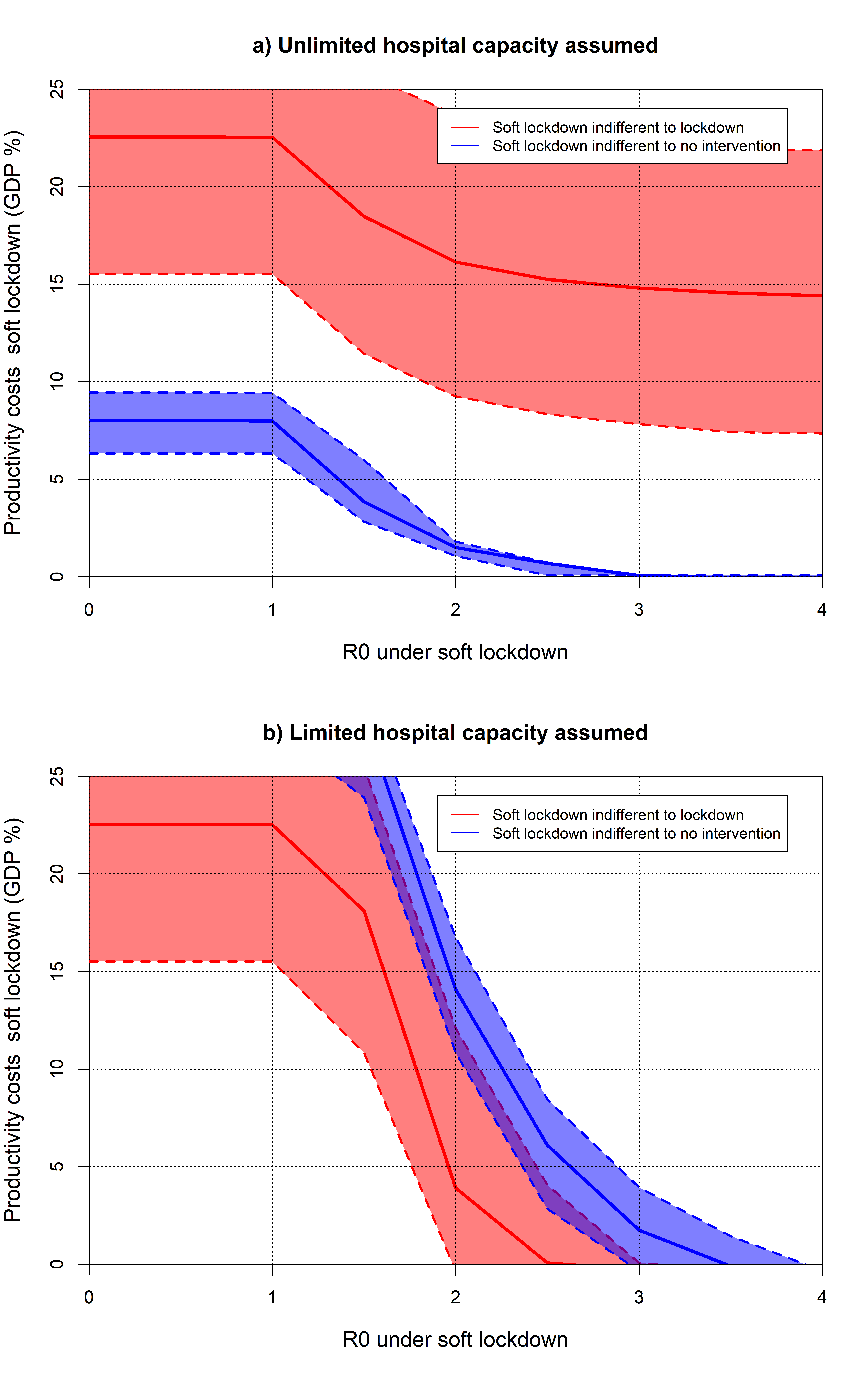


Figure A10. Combinations of $R_{0}$ and GDP losses under soft lockdown that would be indifferent to extending lockdown or no intervention from mid-June 2020. Average and 95% confidence interval (Italy).

**Detailed analysis for Spain**


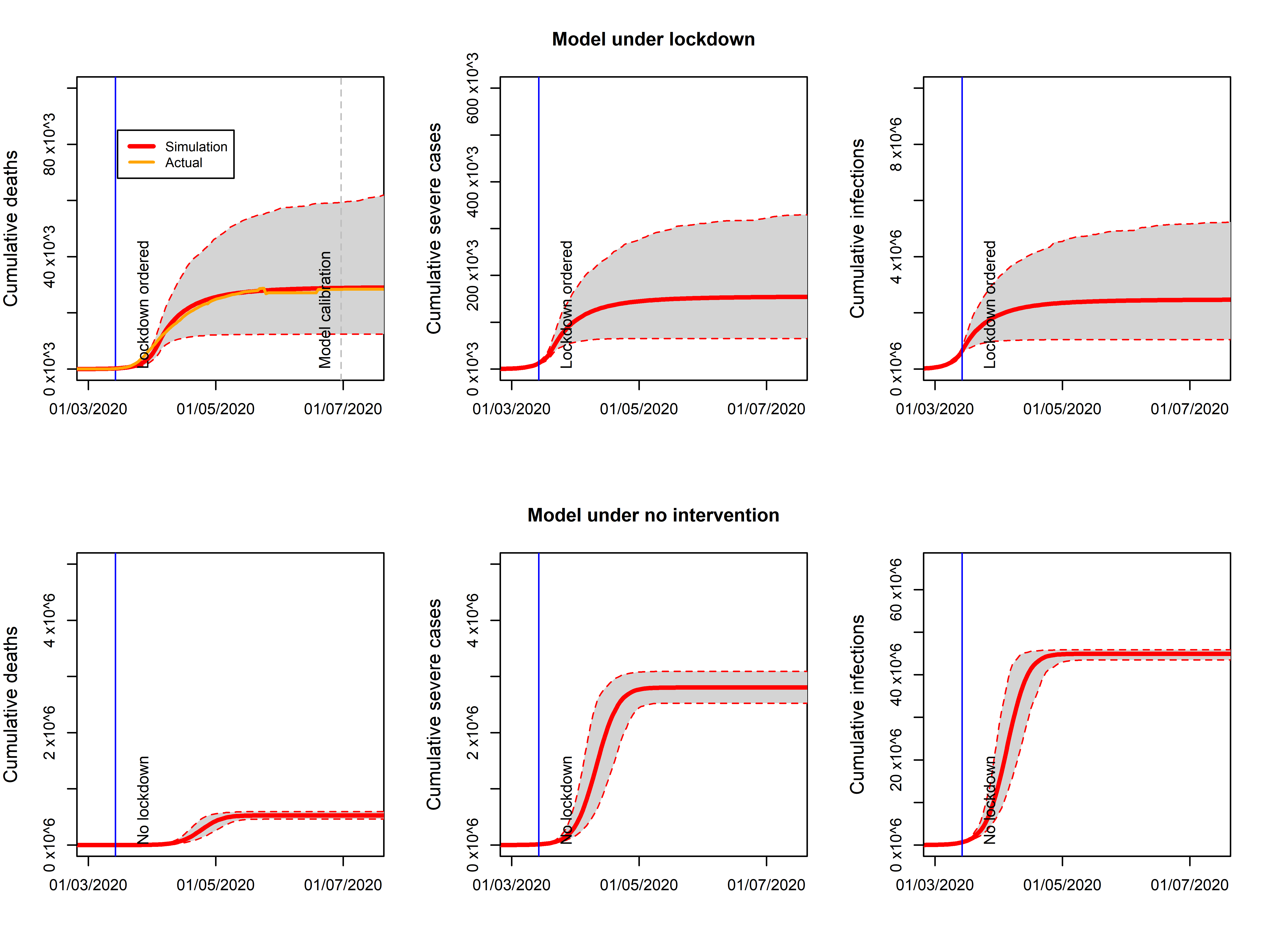


Figure A11. Average and 95% confidence interval of COVID-19 cumulative deaths, hospitalisation demand (severe cases) and infections with/without lockdown. Unlimited hospital capacity assumed (Spain). Note: Mean absolute error between daily new death cases simulated and actual deaths reported was 124.1 between March and June.


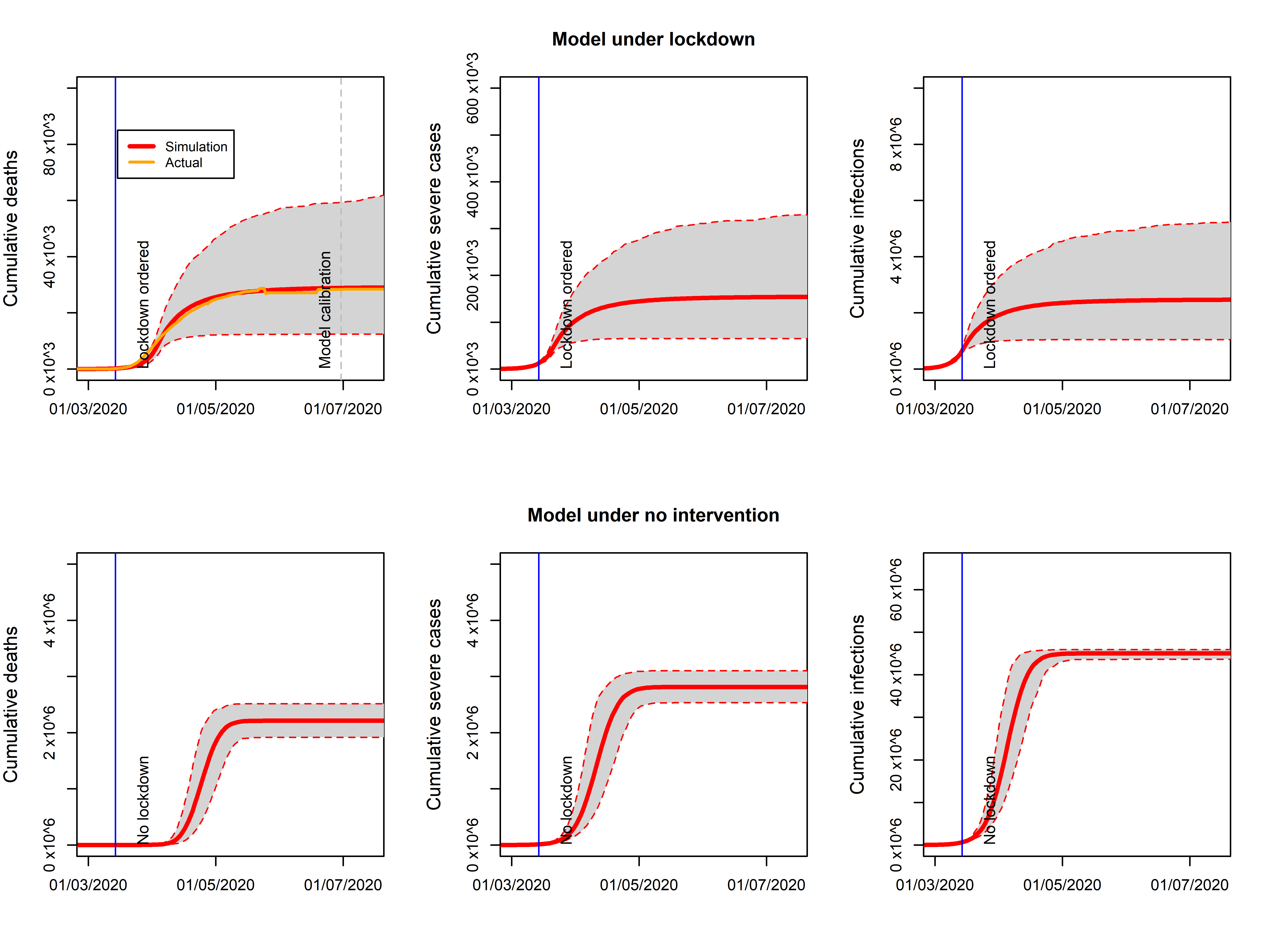


Figure A12. Average and 95% confidence interval of COVID-19 cumulative deaths, hospitalisation demand (severe cases) and infections with/without lockdown. Limited hospital capacity assumed (Spain). Note: Mean absolute error between daily new death cases simulated and actual deaths reported was 124.1 between March and June.


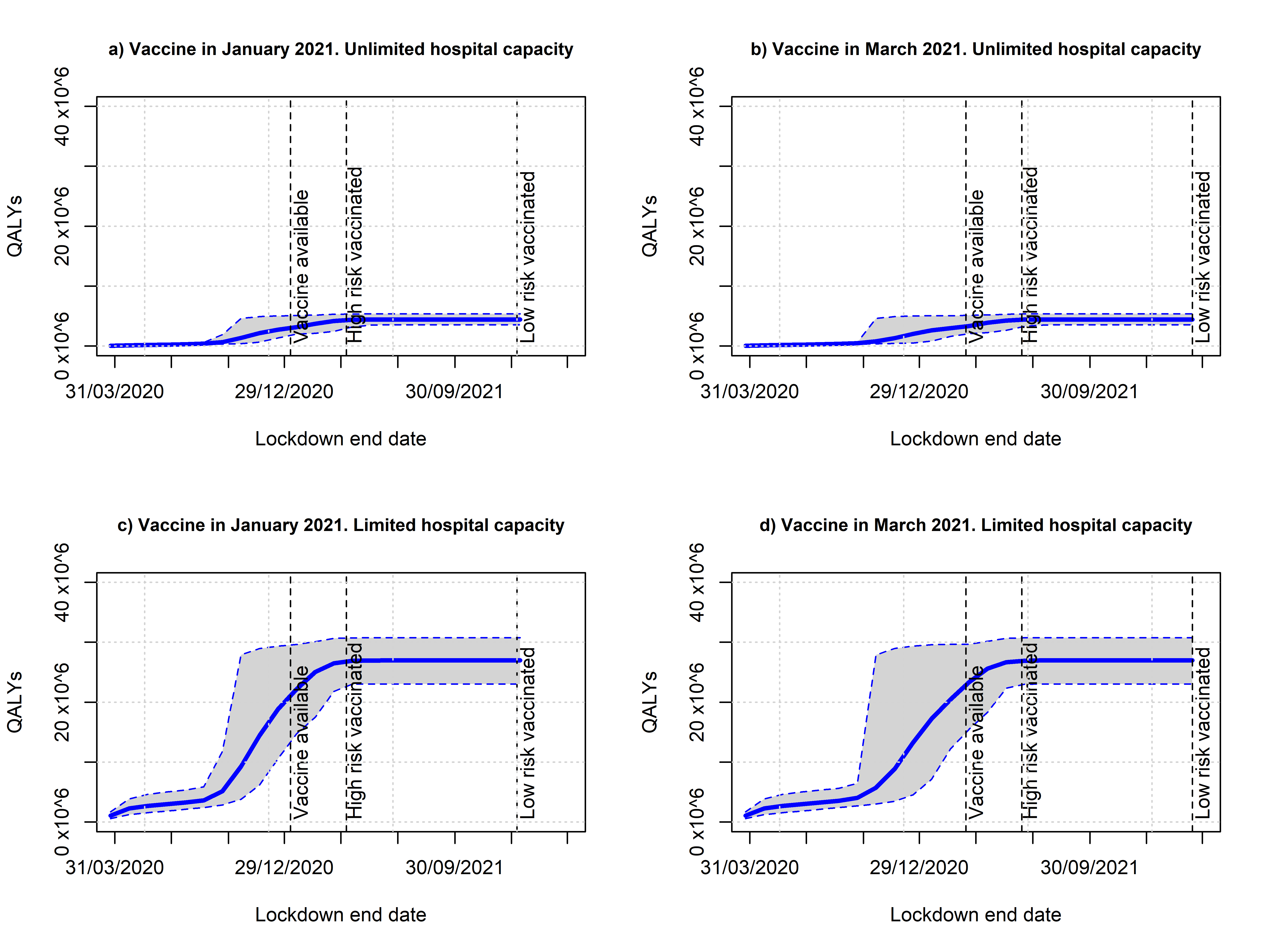
Figure A13. Average and 95% confidence interval of QALYs saved by lockdown duration and time to available vaccine. Unlimited and limited hospital capacity considered (Spain).


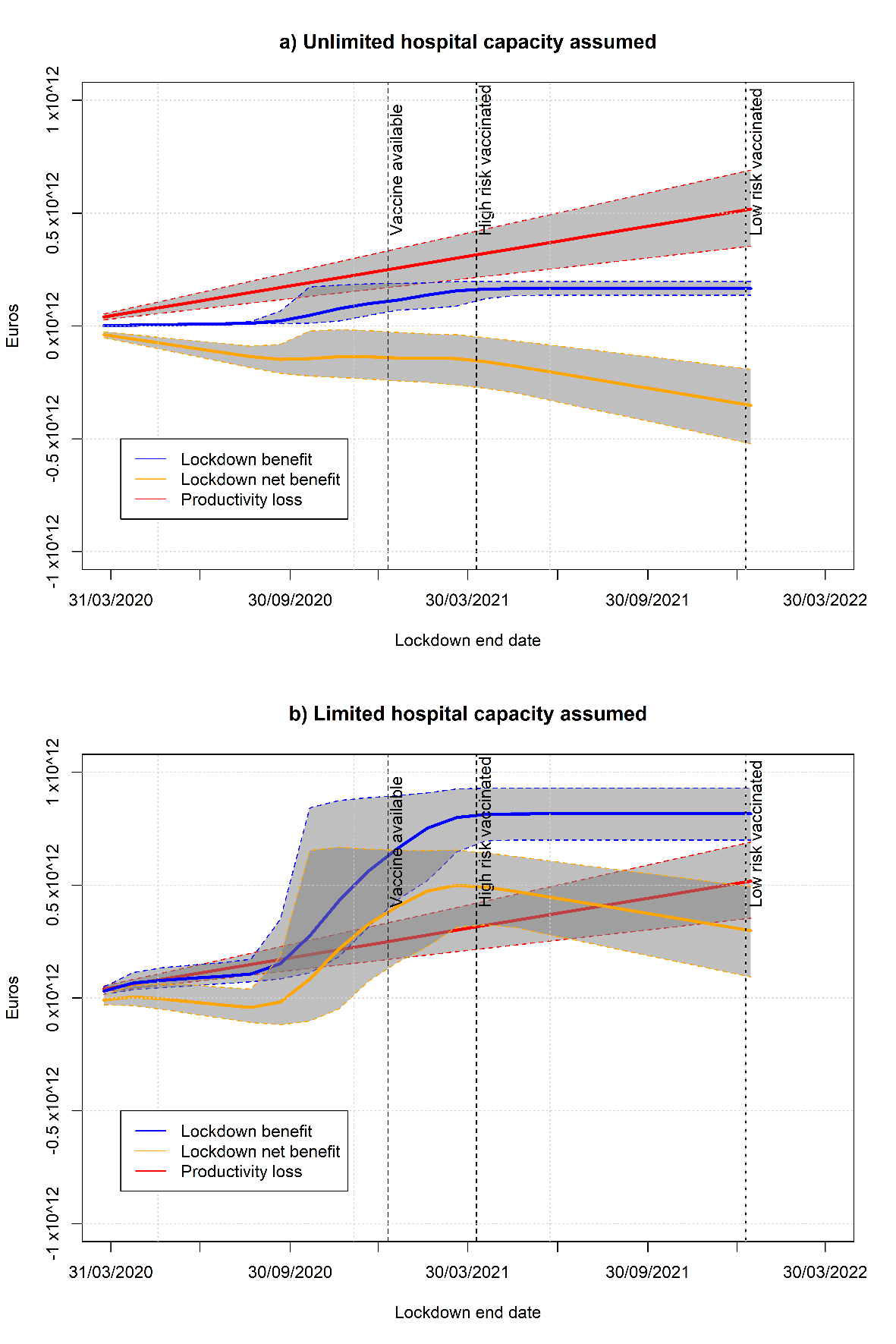


Figure A14. Average and 95% confidence interval of monetary benefit (value of QALYs plus hospitalisations costs saved), productivity losses and net benefit by lockdown duration. Unlimited and limited hospital capacity considered (Spain)


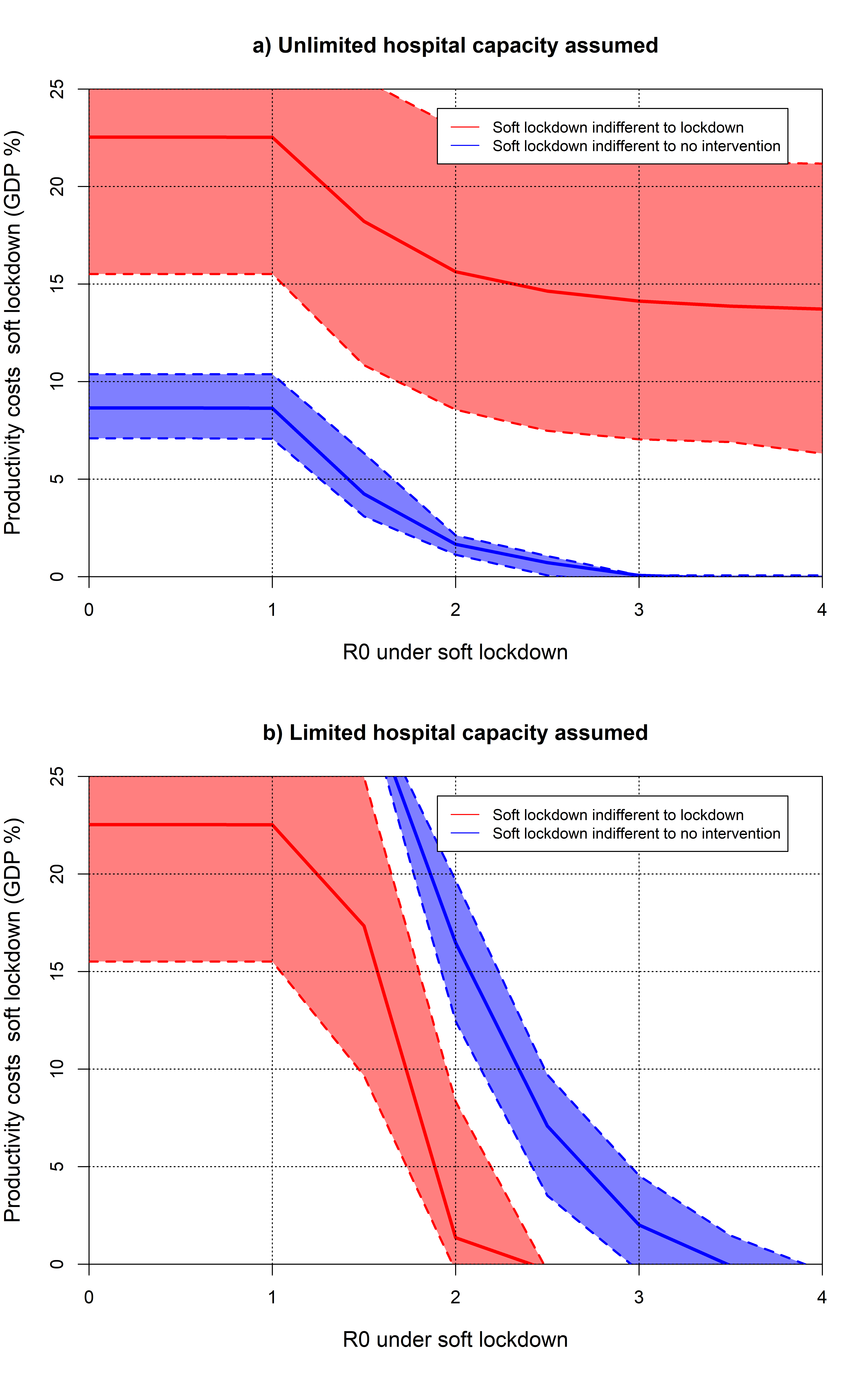


Figure A15. Combinations of $R_{0}$ and GDP losses under soft lockdown that would be indifferent to extending lockdown or no intervention from mid-June 2020. Average and 95% confidence interval (Spain).

REFERENCES

1. Verity R, Okell LC, Dorigatti I, et al. Estimates of the severity of coronavirus disease 2019: a model-based analysis. *The Lancet infectious diseases*. 2020;

2. Neumann-Böhme S, Varghese NE, Sabat I, et al. Once we have it, will we use it? A European survey on willingness to be vaccinated against COVID-19. Springer; 2020.

3. Richiardi M, Bronka P, Collado D. The Economic Consequences of COVID-19 lock-down in the UK. An Input-Output Analysis Using Consensus Scenarios. 2020.

4. Flaxman S, Mishra S, Gandy A, et al. Estimating the effects of non-pharmaceutical interventions on COVID-19 in Europe. *Nature*. 2020;584(7820):257-261.

5. Alimohamadi Y, Taghdir M, Sepandi M. The estimate of the basic reproduction number for novel coronavirus disease (COVID-19): a systematic review and meta-analysis. *Journal of Preventive Medicine and Public Health*. 2020;

6. Jarvis CI, Van Zandvoort K, Gimma A, et al. Quantifying the impact of physical distance measures on the transmission of COVID-19 in the UK. *BMC medicine*. 2020;18:1-10.

7. Bi Q, Wu Y, Mei S, et al. Epidemiology and transmission of COVID-19 in 391 cases and 1286 of their close contacts in Shenzhen, China: a retrospective cohort study. *The Lancet Infectious Diseases*. 2020;

8. Fragaszy EB, Warren‐Gash C, White PJ, et al. Effects of seasonal and pandemic influenza on health‐related quality of life, work and school absence in England: results from the Flu Watch cohort study. *Influenza and other respiratory viruses*. 2018;12(1):171-182.

9. Zhou F, Yu T, Du R, et al. Clinical course and risk factors for mortality of adult inpatients with COVID-19 in Wuhan, China: a retrospective cohort study. *The lancet*. 2020;

10. Linton NM, Kobayashi T, Yang Y, et al. Incubation period and other epidemiological characteristics of 2019 novel coronavirus infections with right truncation: a statistical analysis of publicly available case data. *Journal of clinical medicine*. 2020;9(2):538.

11. Szende A, Janssen B, Cabases J. *Self-reported population health: an international perspective based on EQ-5D*. Springer Netherlands Dordrecht; 2014.

12. Department of Health (2018) NHS reference costs 2017-2018.

13. World Health Organization.  WHO-CHOICE estimates of cost for inpatient and outpatient health service delivery. <https://www.who.int/choice/cost-effectiveness/inputs/country_inpatient_outpatient_2010.pdf?ua=1>
